# Effect of the strain *Bacillus amyloliquefaciens* FZB42 on the microbial community in the rhizosphere of lettuce under field conditions analyzed by whole metagenome sequencing

**DOI:** 10.3389/fmicb.2014.00252

**Published:** 2014-05-27

**Authors:** Magdalena Kröber, Daniel Wibberg, Rita Grosch, Felix Eikmeyer, Bart Verwaaijen, Soumitra P. Chowdhury, Anton Hartmann, Alfred Pühler, Andreas Schlüter

**Affiliations:** ^1^Center for Biotechnology, Institute for Genome Research and Systems Biology, Bielefeld UniversityBielefeld, Germany; ^2^Leibniz-Institute of Vegetable and Ornamental CropsGroßbeeren, Germany; ^3^Research Unit Microbe-Plant Interactions, Helmholtz Zentrum München, German Research Center for Environmental Health (GmbH)Neuherberg, Germany

**Keywords:** metagenome sequencing, taxonomic profiling, *B. amyloliquefaciens* FZB42, lettuce, rhizosphere, fragment recruitment

## Abstract

Application of the plant associated bacterium *Bacillus amyloliquefaciens* FZB42 on lettuce (*Lactuca sativa*) confirmed its capability to promote plant growth and health by reducing disease severity (DS) caused by the phytopathogenic fungus *Rhizoctonia solani*. Therefore this strain is commercially applied as an eco-friendly plant protective agent. It is able to produce cyclic lipopeptides (CLP) and polyketides featuring antifungal and antibacterial properties. Production of these secondary metabolites led to the question of a possible impact of strain FZB42 on the composition of microbial rhizosphere communities after its application. Rating of DS and lettuce growth during a field trial confirmed the positive impact of strain FZB42 on the health of the host plant. To verify *B. amyloliquefaciens* as an environmentally compatible plant protective agent, its effect on the indigenous rhizosphere community was analyzed by metagenome sequencing. Rhizosphere microbial communities of lettuce treated with *B. amyloliquefaciens* FZB42 and non-treated plants were profiled by high-throughput metagenome sequencing of whole community DNA. Fragment recruitments of metagenome sequence reads on the genome sequence of *B. amyloliquefaciens* FZB42 proved the presence of the strain in the rhizosphere over 5 weeks of the field trial. Comparison of taxonomic community profiles only revealed marginal changes after application of strain FZB42. The orders *Burkholderiales, Actinomycetales* and *Rhizobiales* were most abundant in all samples. Depending on plant age a general shift within the composition of the microbial communities that was independent of the application of strain FZB42 was observed. In addition to the taxonomic profiling, functional analysis of annotated sequences revealed no major differences between samples regarding application of the inoculant strain.

## Introduction

Phytopathogenic organisms are a serious threat to food production and storage affecting crop plants and vegetables. Crop losses of approximately 20% and more were caused by plant pathogens worldwide depending on the particular crop (Oerke, 2006). Crop rotation, breeding of resistant cultivars and the use of chemical pesticides are the preferred approaches to reduce the effects of phytopathogens. However, these strategies are insufficient, especially to control root diseases caused by soil-borne plant pathogens of economically important crops (Johri et al., 2003). Additionally, intensive crop management practices increase the problems concerning accumulation of pathogens in the soil (Chellemi, 2002). The facts that excessive use of chemical pesticides caused several environmental problems like soil pollution (Asaka and Shoda, 1996) and that society is becoming more conscious about the environment support the development of alternative control strategies worldwide (Leistra and Matser, 2004; Wang et al., 2004; Alabouvette et al., 2006). Currently, it is well documented that the treatment of plants with plant-associated microorganisms can be effective to decrease the negative impact brought about by infections of the plants by pests (Kazempour, 2004; Scherwinski et al., 2008; Andrews et al., 2012).

Several products based on the application of plant-associated bacteria such as *Bacillus* spp. are commercially available (Choudhary and Johri, 2009). The ability of members of the Gram-positive genus *Bacillus* to form spores is advantageous for preparation of bacterial formulations, storage and product shelf life (Tiago et al., 2004). The effectiveness of these strains relies on different mechanisms. Efficient strains colonize the root system of the host plant, promote plant growth and are able to activate their defense system. This phenomenon of triggering the systemic resistance reaction of the plant has been termed rhizobacteria-induced systemic resistance (ISR) (Arguelles-Arias et al., 2009). Another mechanism that contributes to plant protection is the secretion of different secondary metabolites with antibacterial or antifungal impact on phytopathogenic organisms. In addition, the competition for nutrients and especially iron between biocontrol strains and pathogens is an important factor in the protection of the plant (Arguelles-Arias et al., 2009).

*Bacillus amyloliquefaciens* FZB42 is known for its plant growth promoting properties and its ability to suppress different plant pathogens (Grosch et al., 1999; Idriss et al., 2004; Koumoutsi et al., 2007). Pot and field experiments demonstrated that the strain FZB42 is able to effectively colonize the rhizosphere of lettuce during host plant cultivation and promotes significant suppression of bottom rot disease caused by *Rhizoctonia solani* (Chowdhury et al., 2013). High rhizosphere competence referring to the survival and rhizosphere colonization potential (Bulgarelli et al., 2013) is a key factor for a successful improvement of plant traits and control of plant pathogens by plant-protective agents (Lugtenberg and Kamilova, 2009). Although the commercially available strain FZB42 is a beneficial bacterium, its application may have a negative impact on the indigenous microbial rhizosphere community and associated important ecological functions (Winding et al., 2004). Genome analyses of *B. amyloliquefaciens* revealed that the organism harbors eight different gene clusters comprising genes associated with the production of secondary metabolites providing it with its biocontrol properties (Chen et al., 2007). These secondary metabolites can be classified into three different categories: the polyketides macrolactin (*mln*), bacillaene (*bae*), and difficidin (*dfn*) featuring antibacterial properties (Chen et al., 2006), the cyclic lipopeptides surfactin (*srf*), fengycin (*fen*), and bacillomycin (*bmy*) providing mainly antifungal properties (Koumoutsi et al., 2004) and the third category including the iron-siderophore bacillibactin (*bac*) and a putative siderophore the synthesis of which is encoded by the *nrs* operon.

Due to the high potential of producing secondary metabolites and their possible impact on other microorganisms within the microbial community in the rhizosphere of host plants, the complex interaction between an inoculant strain and the indigenous microbial rhizosphere community has to be analyzed. Knowledge regarding the microbial ecology of the target habitat “rhizosphere” is required for reasonable risk assessment studies related to the application of beneficial plant associated bacterial strains.

To increase the knowledge about the interaction of the strain FZB42 with indigenous rhizosphere community members of lettuce, field trials were carried out at natural occurring pathogen pressure in the field and at higher pathogen pressure achieved by inoculation of the bottom rot pathogen *R. solani* at the same field site (Chowdhury et al., 2013). During the growth period of lettuce whole community DNA samples were isolated from the rhizospheres of lettuce that either were treated with the strain FZB42 (1 week before planting and 4 days after planting) or not treated. Samples from both experiments were collected at planting, 2 weeks and 5 weeks after planting. Analysis of DNA samples from these field trials after 2 and 5 weeks of cultivation of the host plants by 16S rRNA gene amplicon terminal restriction fragment length polymorphism (T-RFLP) revealed no major impact on the general composition of the microbial community regarding the application of the inoculant strain. However, a temporal shift independent from the application of strain FZB42 was observed (Chowdhury et al., 2013).

In contrast to T-RFLP analyses, metagenome studies by means of whole community DNA extraction, high-throughput sequencing and various kinds of sequence analyses applying bioinformatic tools are exceptionally convenient strategies to provide an extended insight into the whole microbial community within a habitat e.g., the rhizosphere. MG-RAST is an established platform to analyze and compare metagenome sequence data in several approaches (Meyer et al., 2008). Within MG-RAST, taxonomic profiling of the community based on metagenome sequences is performed using the NCBI GenBank database comprising sequences of more than 380,000 species. It therefore provides an exceptionally suitable basis for the annotation of metagenome sequences (Benson et al., 2011). Additionally to taxonomic classifications, the MG-RAST software platform provides the possibility to compare functional subsystems based on the SEED resource (http://www.theSEED.org), (Aziz et al., 2008; Overbeek et al., 2014).

To obtain deeper and more refined insights into microbial rhizosphere communities of lettuce, the same DNA samples as for the T-RFLP analysis described above (Chowdhury et al., 2013) were used in a comparative whole metagenome sequencing approach to follow up changes in the taxonomic community profiles during the growth period of lettuce and to identify dominant rhizosphere microorganisms. In addition to the samples used in the 16S rRNA T-RFLP analysis, DNA samples isolated from the rhizosphere at planting 1 week after the first application of strain FZB42 were analyzed by metagenome sequencing in order to reveal possible shifts in the microbial community within the first 2 weeks of cultivation in the field. Previous field trials showed that during this time infections with the phytopathogen *R. solani* are most likely to occur (Grosch et al., 2004).

Another objective of this study was to elucidate the competitiveness and survivability of the inoculant strain FZB42 within the bacterial community in the course of host plant cultivation. The ability of *B. amyloliquefaciens* FZB42 to successfully colonize the rhizosphere is an assumption for reducing disease severity (DS) caused by phytopathogens (Lugtenberg and Kamilova, 2009; Barret et al., 2011; Ghirardi et al., 2012). Expression of genes conferring antifungal activity often is regulated in a cell-density dependent manner (Steidle et al., 2002) and hence corresponding microorganisms should be highly competent in rhizosphere colonization to exert antifungal activity. Proving the establishment of this strain in the rhizosphere of lettuce therefore is the prerequisite for the following analyses addressing alterations in the community profiles between with FZB42 treated and non-treated samples. Therefore, fragment recruitments were performed in which metagenome sequence reads were mapped to the known genome sequence of *B. amyloliquefaciens* FZB42 under strict settings in order to track the occurrence of this strain in the rhizosphere.

In addition to taxonomic profiling, whole metagenome sequencing also allows functional characterization of the rhizosphere community. Studies of microbial rhizosphere communities of different plant species showed that their roots are colonized by only a few dominating phyla (Bulgarelli et al., 2013; Chaparro et al., 2013). These show a common set of metabolic functions connected to genes involved in carbohydrate, nitrogen and amino acid metabolism. This observation has led to the assumption that specific functions are essential for a successful colonization of this habitat during the growth of the plants. The treatment of plants with *B. amyloliquefaciens* FZB42 and its secretion of secondary metabolites with antimicrobial properties potentially may lead to shifts within rhizosphere microbial functional subsystems affecting e.g., availability of nutrients for the plant and other important properties in the rhizosphere. Furthermore, the functional subsystem of virulence and defense mechanisms was analyzed since alterations within this category may be caused by application of the inoculant strain FZB42.

## Materials and methods

### Field trial and preparation of DNA samples

A field trial was carried out at the Institute of Vegetable and Ornamental Crops (Golzow, Germany, 52° 34′ N, 14° 30′ E) to determine the ability of *B. amyloliquefaciens* FZB42 to colonize the rhizosphere of lettuce plants (cv. Tizian, Syngenta, Bad Salzuflen, Germany) and the potential impact of the treatment with this strain on the composition of the microbial community within this habitat. Prior to the cultivation in the field the young plants were grown in peat blocks at 20/15°C (16/8 h, day/night cycle) and were then transferred in the 3–4 leaf state to the field with alluvial loam (total N 112; P 32.3; K 17.4; and Mg 9.1 mg/100 g soil; pH 6.5). Lettuce plants were first treated with FZB42 in the 2–3-leaf stage 1 week before planting into the field beds. Each seedling tray with 150 plants was watered with 1.74 l spore suspension (10^7^ CFU/ml) of FZB42. Four days after planting, the lettuce plants at the 3–4-leaf stage were treated with a spore suspension (10^7^ CFU/ml) of FZB42 for a second time. An amount of 0.5 l spore suspension was applied by hand sprayer to each bed with the size of 6.75 m^2^ with 11 plants per m^2^. The experiment comprised non-treated (control) and treated (inoculated) plants at planting (t0), 2 weeks (t1) and 5 weeks (t2) after planting of lettuce into the field. Total community DNAs were isolated from the rhizosphere of three lettuce plants per replicate and combined prior to DNA isolation. Samples were taken from four replicates randomly arranged in the experimental field. This approach resulted in the analysis of 12 plants per treatment and time point. The experimental design and sampling regime of this study is outlined in detail in a previous publication (Chowdhury et al., 2013).

### High-throughput sequencing of metagenomic DNA

Whole metagenome shotgun libraries were constructed based on the metagenomic DNA preparations extracted from the rhizosphere of lettuce. Prior to library preparation 3.3 ng DNA from three replicates (six plants each) were pooled per treatment and sampling time point. Accordingly, each metagenomic library comprises rhizosphere total community DNA preparations from 12 plants per treatment. Sequencing of these libraries was performed on the MiSeq system (Illumina) according to protocols provided by Illumina.

### Bioinformatic analysis of metagenome sequence data

Metagenome reads were processed and analyzed within the MG-RAST software package (Meyer et al., 2008; Wilke et al., 2013). Sequence data are available from the MG-RAST platform under the following IDs: t0 control: http://metagenomics.anl.gov/linkin.cgi?metagenome=4535147.3, t0 inoculated: http://metagenomics.anl.gov/linkin.cgi?metagenome=4535148.3, t1 control: http://metagenomics.anl.gov/linkin.cgi?metagenome=4535149.3, t1 inoculated http://metagenomics.anl.gov/linkin.cgi?metagenome=4535150.3, t2 control: http://metagenomics.anl.gov/linkin.cgi?metagenome=4535151.3, t2 inoculated: http://metagenomics.anl.gov/linkin.cgi?metagenome=4535152.3.

Taxonomic profiling and determination of α-diversity was performed within MG-RAST applying the “Reprehensive Hit Classification” option using the GenBank database as reference with the following settings: maximum e-value cut off of 1^*^10^−5^, minimum identity of 80%, and minimum alignment length of 15 measured in amino acids for proteins and base pairs for RNA databases. Taxonomic information was inferred from the best BLAST hit applying the “Representative Hit” option within MG-RAST. Compositions of microbial communities were compared based on normalized ratios for each taxon identified within a specific dataset. Ratios of identified taxa were calculated by referring them to the total amount of classified sequences within each dataset. The deduced taxonomic profiles were visualized by means of Microsoft Excel (2008). Additionally, MA-plots were calculated. These plots visualize the distribution of annotated genera within control and inoculated sample sets by plotting the logarithmic ratios between the numbers of annotated sequences (*M*-value) against the logarithmic mean average of annotated sequences (*A*-value) of each genus.

### Annotation of functional subsystems

Analysis of the relevant functional subgroups was performed using the SEED subsystems technology within the MG-RAST software package with default settings. Proteins deduced from metagenome reads were compared to the FIGfam protein family collection within MG-RAST and then classified according to the predefined subsystems applying the SEED technology implemented in MG-RAST (Aziz et al., 2008; Meyer et al., 2008; Overbeek et al., 2014). All metagenome sequences matching a specific subsystem were counted and this value was then divided by the number of sequences that were assigned to any subsystem to give fractions and therefore normalized, comparable values (Meyer et al., 2008).

### Mapping of metagenome sequence reads to the *B. amyloliquefaciens* FZB42 genome

Metagenome sequence reads of the six datasets were mapped to the *B. amyloliquefaciens* FZB42 reference genome (GenBank Accession: CP000560) by means of the gsMapper program (Roche Genome Analyzer Data Analysis Software Package, version 2.8) in order to follow the occurrence of the inoculant strain in the rhizosphere. Strict settings of the gsMapper (98% sequence identity, minimum 225 bp length, minimum 90% overlap) were used for this fragment recruitment to specifically map reads originating from *B. amyloliquefaciens* FZB42. Multiple contigs and corresponding consensus sequences were generated from the mapped reads. To visualize the positions of the mapped reads in the *B. amyloliquefaciens* FZB42 genome, a Circos plot of the resulting contigs for each time point was calculated (Krzywinski et al., 2009). For the determination of the traceable amounts of strain FZB42 in the course of the cultivation, the normalized numbers of metagenome reads only matching specific *B. amyloliquefaciens* FZB42 genes were used.

## Results

### Metagenome sequencing of lettuce rhizosphere microbial communities

**Figure 1:**
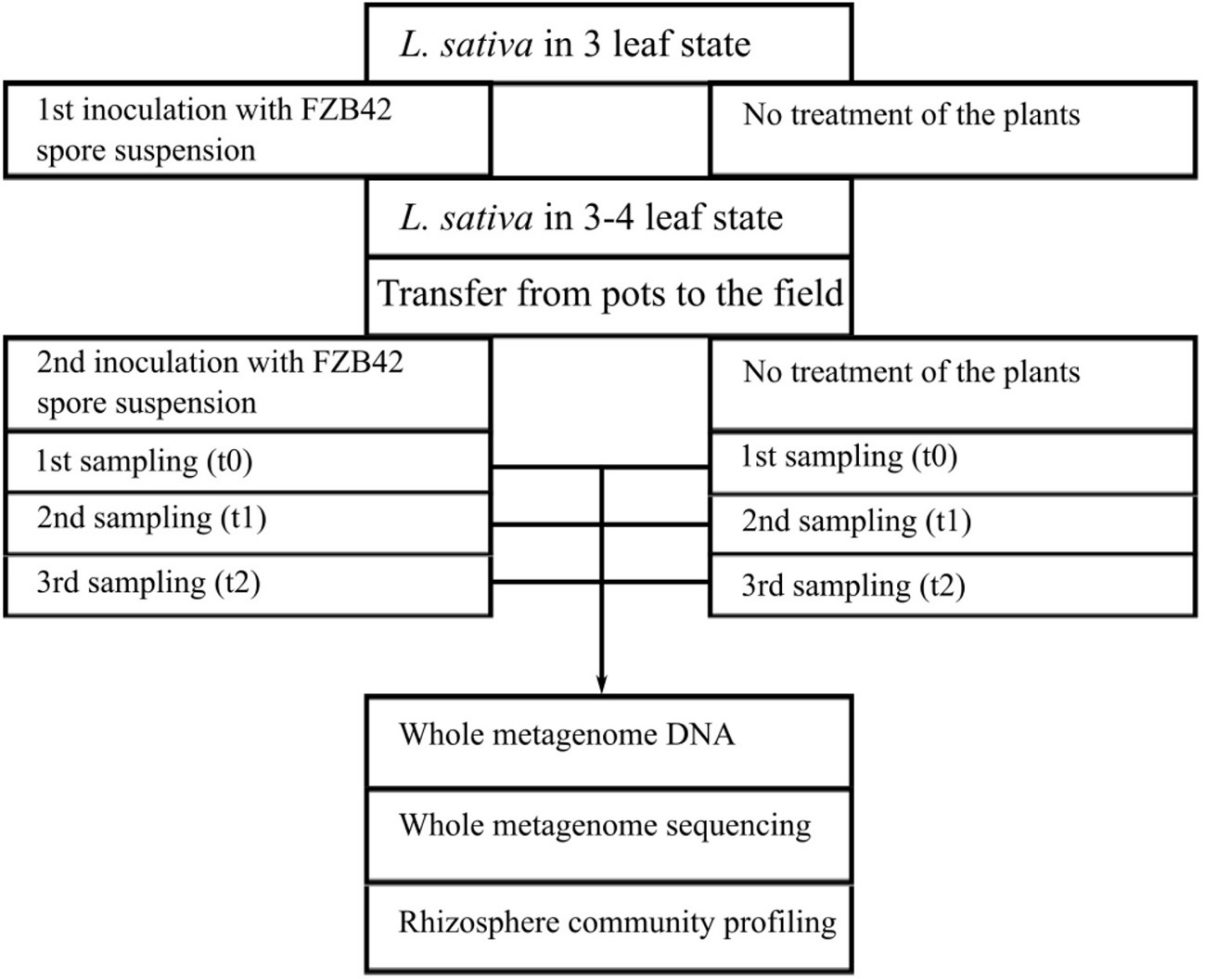
**Scheme of the lettuce field cultivation experiment**. Lettuce was either inoculated with *B. amyloliquefaciens* FZB42 or not treated. Samples of both experiments were drawn at planting (t0), 2 weeks (t1), and 5 weeks (t2) of cultivation from the rhizosphere of the inoculated and non-treated plants. Rhizosphere samples were used for whole community DNA extraction and subsequently for metagenome sequencing. Annotated metagenome reads were used for taxonomic and functional profiling of the microbial communities within the lettuce rhizospheres.

To determine the capability of *B. amyloliquefaciens* FZB42 to colonize the lettuce rhizosphere, taxonomic and functional profiles of microbial rhizosphere communities were deduced from corresponding metagenome sequence data. Since in the previous study (Chowdhury et al., 2013) comparison of T-RFLP patterns obtained for replicates of rhizosphere DNA preparations revealed consistent results over the replicates per sample, it was decided to pool the rhizosphere samples per treatment and sampling time point in the metagenome study described here. Table 1 shows the results of the metagenome sequencing approach of six different DNA-libraries from a field trial that schematically is represented in Figure 1.

**Table 1:** **Statistics of metagenome sequencing of DNA preparations from the rhizosphere of *Lactuca sativa* treated with *B. amyloliquefaciens* FZB42 (+FZB42) and corresponding controls**.

	**t0 control**	**t0 inoculated**	**t1 control**	**t1 inoculated**	**t2 control**	**t2 inoculated**
Sequences	1,909,294	2,500,586	799,247	1,510,665	1,590,425	995,370
High quality sequences	1,869,056	2,446,824	783,195	1,470,887	1,550,845	971,074
Mean sequence length (bp)	218 ± 52	218 ± 53	220 ± 52	213 ± 55	215 ± 54	216 ± 54
Mean GC content (%)	58 ± 12	58 ± 11	62 ± 10	62 ± 10	61 ± 11	60 ± 12
Annotated sequences	624,429	706,259	169,574	286,817	344,059	203,447
Annotated *Bacteria*	614,382	695,861	166,878	281,151	338,012	198,840

Yields of the sequencing runs were determined to be between 783,195 and 2,446,824 reads after quality control (Table 1). Taxonomic annotation with the implemented “Representative Hit Classification” of MG-RAST against the GenBank database resulted in the annotation of between 169,574 and 706,259 sequence reads considering all taxonomic domains. Classified reads represent between 19.5 and 33.4% of all sequences. Among all annotated taxonomic domains 98.2% of the reads were assigned to the domain *Bacteria.* (Table 1).

### Estimation of lettuce rhizosphere colonization by *B. amyloliquefaciens* FZB42 based on fragment recruitment analyses

**Figure 2:**
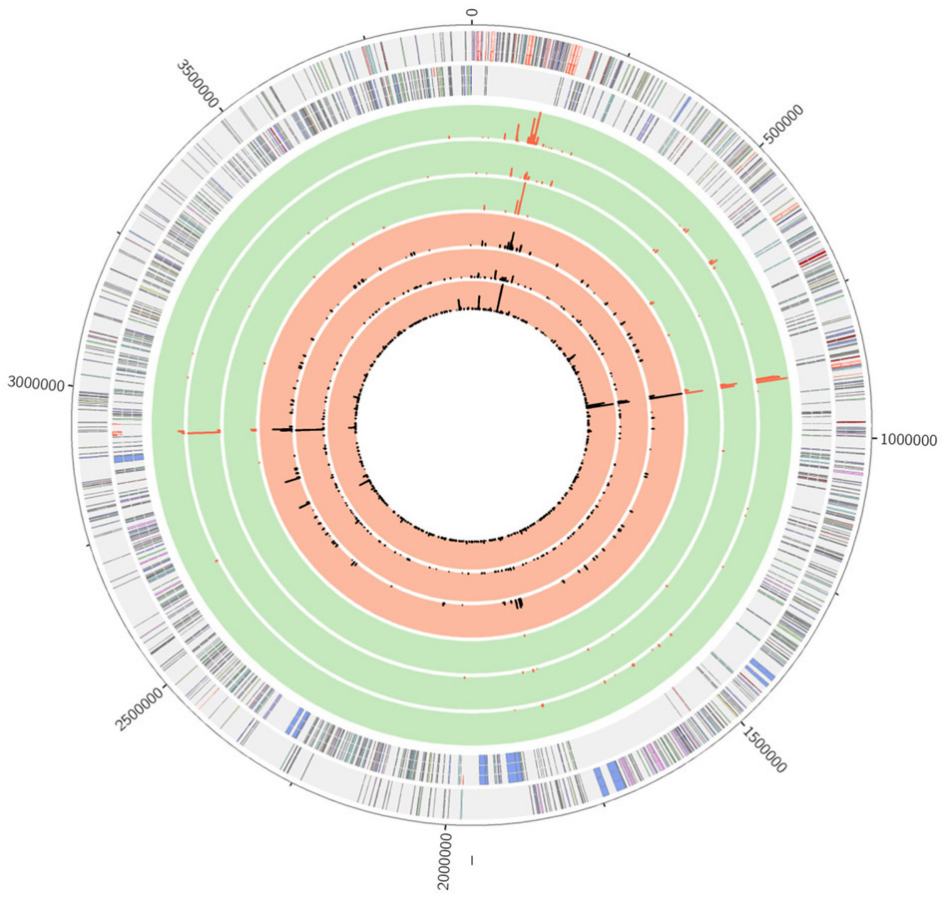
**Mapping of metagenome sequence reads to the *B. amyloliquefaciens* FZB42 genome**. The circles represent from inner to outer most: (i) t0 inoculated sample; (ii) t1 inoculated sample; (iii) t2 inoculated sample; (iv) t0 control; (v) t1 control; (vi) t2 control; (vii) annotated coding sequences in both strands; (viii) scale in bp. The replication initiation gene *dnaA* was chosen as first gene of the chromosome. Coding sequences are colorized depending on their gene function according to COG (Clusters of Orthologous Groups of proteins) categories. The scale for mapped reads represents the relative abundance of reads per base normalized to the maximum value of mapped reads per base.

Prior to taxonomic profiling and comparison of community profiles, metagenome sequences were used to estimate rhizosphere colonization of the inoculant strain in order to relate potential changes within the taxonomic and functional profiles to the application of *B. amyloliquefaciens* FZB42. Mapping of the metagenome sequences to the reference genome of *B. amyloliquefaciens* FZB42 revealed that the strain is able to colonize the rhizosphere of lettuce over the course of the whole growth period (Figure 2 and Table 2).

**Table 2:** **Mapping of reads, contigs and genes from the metagenome datasets to the *B. amyloliquefaciens* FZB42 reference genome**.

	**t0 control**	**t0 inoculated**	**t1 control**	**t1 inoculated**	**t2 control**	**t2 inoculated**
Mapped reads^a^	7,859	10,143	2,808	5,539	5,251	3,478
Mapped reads (%)^b^	0.21	0.20	0.18	0.18	0.17	0.18
Number of contigs^c^	28	619	48	256	75	155
Contig size [bp]^d^	5,957	203,115	9,038	80,095	13,421	44,859
Covered genes^e^	25	598	58	266	74	162

a Number of metagenome sequence reads mapped to the FZB42 reference genome.

b Ratio of mapped reads within all high quality metagenome sequence reads (see Table 1).

c Number of contigs assembled from mapped reads.

d Resulting contig size of the assembled contigs.

e Number of genes hit by metagenome sequence reads.

Comparison of the amount of mapped reads and the resulting assembled contigs between samples taken at the different time points showed that for the first sample (t0) more reads from inoculated rhizospheres were mapped to the reference genome. Figure 2 clearly shows that within the first inoculated sample a wide set of genes of FZB42 is covered by 10,143 metagenome sequences adding up to a contig size of 203,115 bp. In the course of the experiment, the number of sequences specifically matching the *B. amyloliquefaciens* FZB42 genome decreased considerably to 5539 in the t1 and 3478 in the t2 inoculated sample, but still a part of the reference genome is covered by metagenome sequences. In contrast, metagenome sequence reads from non-inoculated samples mapping to the reference genome accumulate in very small sections of the genome.

In addition to the number of mapped reads to the reference genome, the corresponding annotated genes were determined. The number of FZB42 genes covered by metagenome reads was considerably higher in all inoculated samples compared to the control samples and are distributed over the whole genome (Table 2). Sequence reads from control samples that were mapped to the reference genome mainly represent genes encoding rRNAs and mobile genetic elements, which most probably are widely spread in species of the genus *Bacillus*. Among the completely covered genes for these samples, only 19 were already annotated and either corresponded to rRNA genes or other conserved housekeeping genes or have no assigned function yet. Accordingly, reads recruited to the *B. amyloliquefaciens* FZB42 genome from the metagenome data sets of the control samples most probably originate from indigenous *Bacillus* species that share particular genes or DNA elements with *B. amyloliquefaciens* FZB42. However, 835 *B. amyloliquefaciens* FZB42 genes featured recruited metagenome reads originating only from the inoculated samples. These genes found no matching reads in any control sample. The 835 specific genes were used to trace occurrence of the inoculant strain during the growth period of lettuce. Metagenome sequence reads hitting any of these specific genes with high stringency (settings are given in Material and Methods) were counted for the inoculated samples for the sampling time points t0, t1, and t2 after normalization of datasets (Supplementary table 1). As mentioned above, none of the reads from the control samples matched any gene determined to be specific for *B. amyloliquefaciens* FZB42. It appeared that recruited read counts from with FZB42 treated samples decreased from 100 (t0) to 64% (t1) to 55% at the end of the experiment (t2). In summary, fragment recruitments proved occurrence of the inoculant strain *B. amyloliquefaciens* FZB42 in the rhizosphere of lettuce. However, abundance of the strain declines in the course of the experiment.

### Comparative taxonomic characterization of the microbial communities in the lettuce rhizospheres with and without application of *B. amyloliquefaciens* FZB42 by whole metagenome sequence analyses

Determination of the α-diversities summarizing the mean species diversity in habitats represented by the six metagenome data sets showed a clear increase from 332.82 in the control sample and 349.23 in the with FZB42 treated sample taken after at planting to 430.81 in the control sample and 447.81 in the with FZB42 treated sample taken 2 weeks after planting (Table 3). After 5 weeks, the α-diversities of 412.71 in the control and 437.21 in the inoculated sample were measured.

**Table 3:** **α diversities within the metagenome datasets**.

	**t0 control**	**t0 inoculated**	**t1 control**	**t1 inoculated**	**t2 control**	**t2 inoculated**
α diversity	332.82	349.23	430.81	447.14	412.71	437.21

On average, the reads of the sequenced metagenome libraries were assigned to 27 phyla, 41 classes, 92 orders, 211 families, and 596 genera within the superkingdom *Bacteria* (Table 4). Considering the six samples for different treatments and the three sampling time points, a total of 27 phyla, 41 classes, 95 orders, 217 families, and 689 genera were detected. Deduced taxonomic profiles clearly overlap at higher taxonomic levels and in a slightly decreasing manner at lower levels for the different samples. These results indicate that the general compositions of the analyzed microbial communities within the lettuce rhizospheres are similar.

**Table 4:** **Numbers of different taxa assigned to the superkingdom *Bacteria* detected within the metagenome sequences as analyzed by the MG-RAST software package**.

	**t0 control**	**t0 inoculated**	**t1 control**	**t1 inoculated**	**t2 control**	**t2 inoculated**	**sum^a^**
Phylum	27	27	27	27	27	27	27
Class	41	41	41	41	41	41	41
Order	92	93	91	92	92	91	95
Family	210	213	211	214	210	210	217
Genus	602	613	588	601	598	573	689

a Combined number of different taxa within all samples.

**Figure 3:**
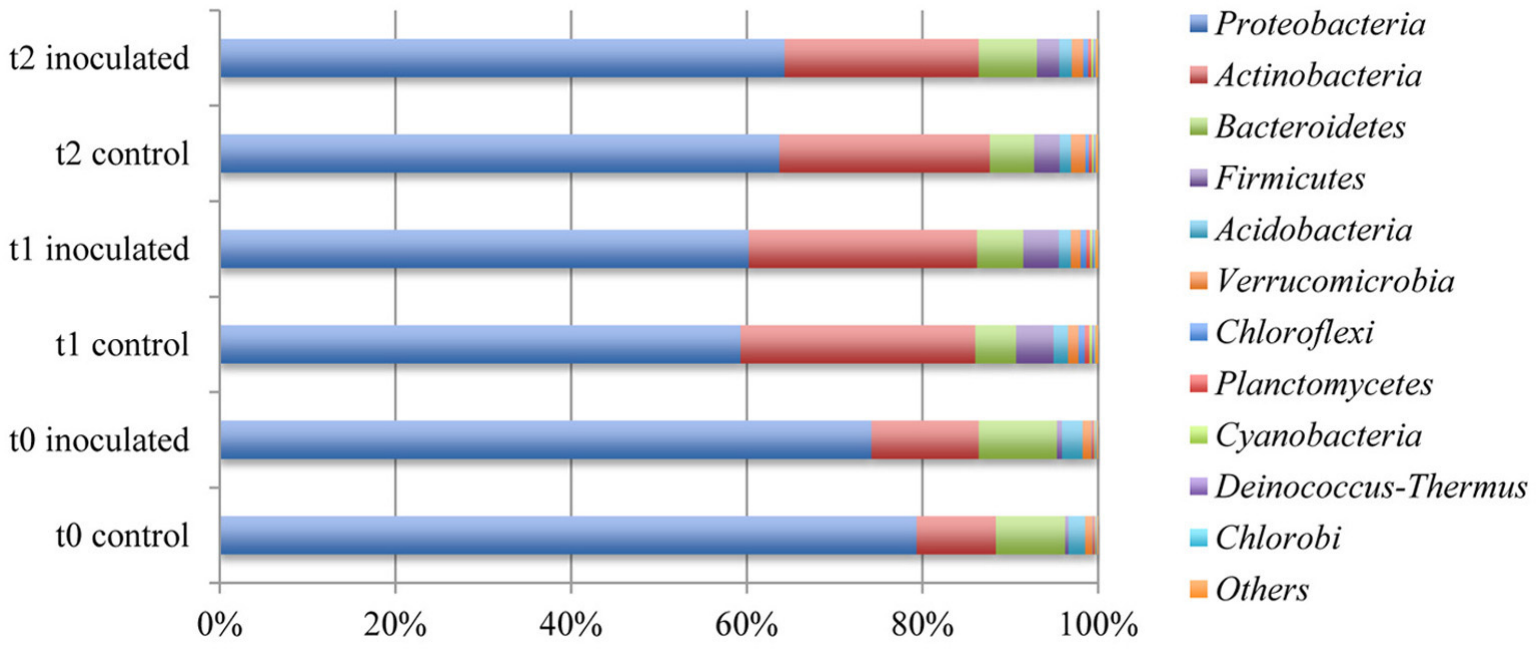
**Comparison of the ten most abundant phyla within the rhizosphere of lettuce**. Abundances were calculated by means of comparison of metagenome sequence reads against the GenBank database within the MG-RAST software package.

Analysis of the most abundant phyla within the sample sets taken at different time points revealed a high overlap resulting in only 11 phyla representing the 10 most prominent phyla within the single sample sets. The most abundant phyla *Proteobacteria*, *Actinobacteria*, *Bacteroidetes*, and *Firmicutes* represented 95.0 to 96.7% of all assigned sequences (Table 5a and Figure 3). Within the metagenome datasets derived from the control samples and samples from inoculated rhizospheres, only minor changes are noticeable, whereas a more distinct shift between relative frequencies of assigned community taxa in the course of the experiment can be observed. The most obvious changes were detectable within the mentioned three most prominent phyla of the microbial communities after 2 weeks of cultivation in the field. The amount of annotated *Proteobacteria* decreased from over 74% to approximately 59% in the t1 samples to 64% in the t2 samples, and *Bacteroidetes* decreased from around 8% (t0) to about 4.5% (t1) to 6.6% (t2). The ratios of annotated *Actinobacteria* increased from between 9% in the control sample and 12% in the with FZB42 treated sample from t0 to around 24% within the samples taken after 2 and 5 weeks. Additionally the ratio of *Firmicutes* increased from around 0.4% in the t0 samples to around 4% within the t1 and then decreased again to around 2.7% in the t2 samples. The remaining frequencies of annotated phyla varied only slightly between all samples.

**Table 5:** **Relative abundances of the dominant bacterial phyla (a), classes (b), orders (c), and genera (d) within the rhizosphere of *L. sativa* calculated for all classified sequences within the bacterial domain**.

	**t0 control**	**t0 inoculated**	**t1 control**	**t1 inoculated**	**t2 control**	**t2 inoculated**
**(a) PHYLUM**						
*Proteobacteria*	79.35	74.23	59.31	60.27	63.74	64.33
*Actinobacteria*	9.03	12.21	26.74	25.98	23.95	22.12
*Bacteroidetes*	7.88	8.92	4.65	5.26	5.04	6.62
*Firmicutes*	0.39	0.56	4.27	4.07	2.92	2.54
*Acidobacteria*	1.90	2.37	1.63	1.34	1.28	1.42
*Verrucomicrobia*	0.82	0.98	1.20	1.13	1.65	1.31
*Chloroflexi*	0.08	0.09	0.73	0.64	0.38	0.55
*Planctomycetes*	0.18	0.22	0.53	0.43	0.36	0.37
*Cyanobacteria*	0.11	0.13	0.30	0.25	0.20	0.25
*Deinococcus-Thermus*	0.06	0.08	0.22	0.19	0.12	0.14
*Chlorobi*	0.07	0.09	0.10	0.13	0.10	0.11
Others	0.12	0.14	0.33	0.33	0.26	0.25
**(b) CLASS**						
*Alphaproteobacteria*	24.49	27.14	25.38	26.11	31.61	31.73
*Betaproteobacteria*	30.61	29.62	20.63	23.88	20.42	20.54
*Actinobacteria*	9.07	12.27	26.99	26.18	24.10	22.26
*Gammaproteobacteria*	23.99	17.10	11.63	8.49	10.70	10.86
*Sphingobacteria*	4.26	5.15	2.25	2.28	3.27	2.96
*Bacilli*	0.19	0.35	3.55	3.39	2.46	2.03
*Flavobacteria*	2.05	1.91	1.37	1.92	0.88	2.65
*Deltaproteobacteria*	0.59	0.70	2.11	2.12	1.33	1.52
*Cytophagia*	1.35	1.61	0.81	0.86	0.70	0.78
*Acidobacteria*	1.47	1.87	0.47	0.39	0.70	0.66
*Opitutae*	0.43	0.55	0.37	0.35	0.84	0.65
*Clostridia*	0.18	0.19	0.71	0.65	0.43	0.49
Others	1.33	1.55	3.74	3.39	2.57	2.86
**(c) ORDER**						
*Burkholderiales*	28.87	27.98	19.25	22.53	17.85	17.63
*Actinomycetales*	8.63	11.84	25.25	24.66	23.00	21.13
*Rhizobiales*	12.20	14.15	14.29	14.27	19.02	17.58
*Pseudomonadales*	14.54	8.29	5.97	3.28	5.94	5.98
*Caulobacterales*	8.43	8.76	3.94	3.80	5.68	5.82
*Sphingomonadales*	1.84	1.93	5.62	6.41	5.24	6.54
*Xanthomonadales*	4.94	5.17	2.38	2.69	2.65	2.60
*Sphingobacteriales*	4.30	5.21	2.27	2.30	3.32	3.00
*Bacillales*	0.16	0.32	3.51	3.34	2.45	1.99
*Flavobacteriales*	2.03	1.90	1.36	1.91	0.87	2.65
*Enterobacteriales*	3.05	1.94	2.00	1.14	0.75	0.90
*Methylophilales*	1.11	1.07	0.65	0.56	2.08	2.22
Others	9.89	11.44	13.50	13.11	11.16	11.95
**(d) GENUS**						
*Mycobacterium*	3.89	7.42	3.82	3.80	5.67	4.42
*Pseudomonas*	5.55	5.04	3.70	2.20	5.53	5.39
*Burkholderia*	8.07	6.86	2.71	2.66	3.13	2.73
*Caulobacter*	5.10	5.18	2.64	2.37	4.17	4.43
*Acidovorax*	3.28	3.51	2.98	4.09	2.32	2.81
*Variovorax*	1.77	1.82	3.65	4.56	3.19	3.00
*Streptomyces*	0.39	0.47	3.36	3.37	4.47	3.80
*Arthrobacter*	0.69	0.56	5.45	5.44	1.66	1.58
*Rhodopseudomonas*	1.85	2.42	2.22	2.34	3.23	3.19
*Acinetobacter*	8.47	2.73	2.11	0.94	0.16	0.36
*Polaromonas*	2.81	3.25	1.98	2.45	2.14	2.12
*Nocardioides*	1.02	0.76	2.77	2.61	2.87	2.93
*Sphingomonas*	0.61	0.64	2.44	2.78	2.24	2.99
*Xanthomonas*	2.45	2.59	1.18	1.37	1.32	1.37
*Pedobacter*	2.19	2.57	0.93	0.93	1.19	1.22
*Bacillus*	0.05	0.16	2.57	2.48	1.95	1.54
Others	51.82	54.02	55.49	55.60	54.76	56.12

**Figure 4:**
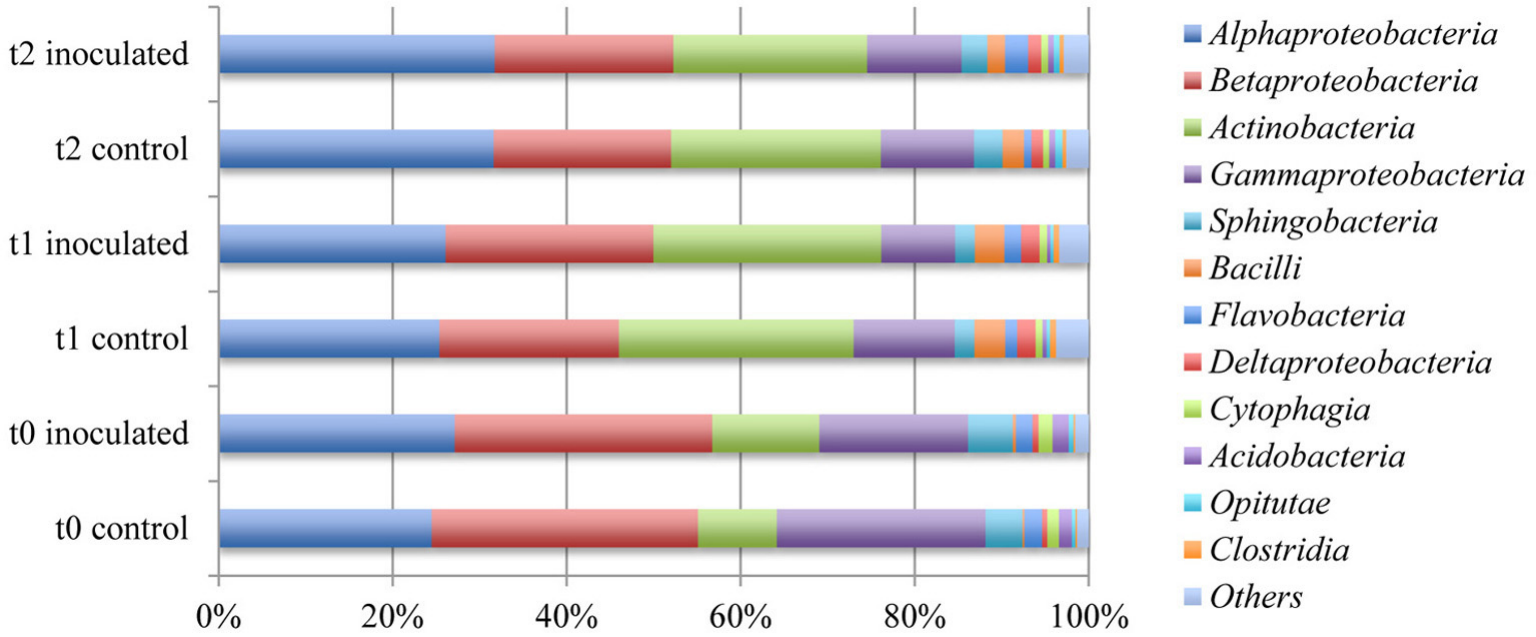
**Comparison of the ten most abundant classes within the rhizosphere of lettuce**. Abundances were calculated by means of comparison of metagenome sequence reads against the GenBank database within the MG-RAST software package.

The 10 most abundant classes of the single data sets add up to 12 different classes represented by around 97.4% of all annotated bacterial sequences. The four most prominent classes *Alphaproteobacteria, Betaproteobacteria*, *Actinobacteria*, and *Gammaproteobacteria* were represented by 84.6–88.2% of all annotated sequences (Table 5b and Figure 4). Distinct changes between samples taken at different time points were detected within the first 2 weeks of cultivation, whereas the microbial rhizosphere communities were relatively stable in the last 3 weeks of cultivation. The relative abundance of annotated *Betaproteobacteria* that was around 30% at t0, decreased to 22% at t1 and around 20% in the t2 samples. The amount of annotated *Actinobacteria* increased noticeably from between 9 and 12% to around 24.8% in average in the samples drawn after 2 and 5 weeks. The relative abundances of annotated *Gammaproteobacteria* varied between the t0 samples between 24% in the control and 17% in the inoculated samples. Within the samples taken after 2 and 5 weeks of cultivation in the field the ratio of *Gammaproteobacteria* was around 10%.

**Figure 5:**
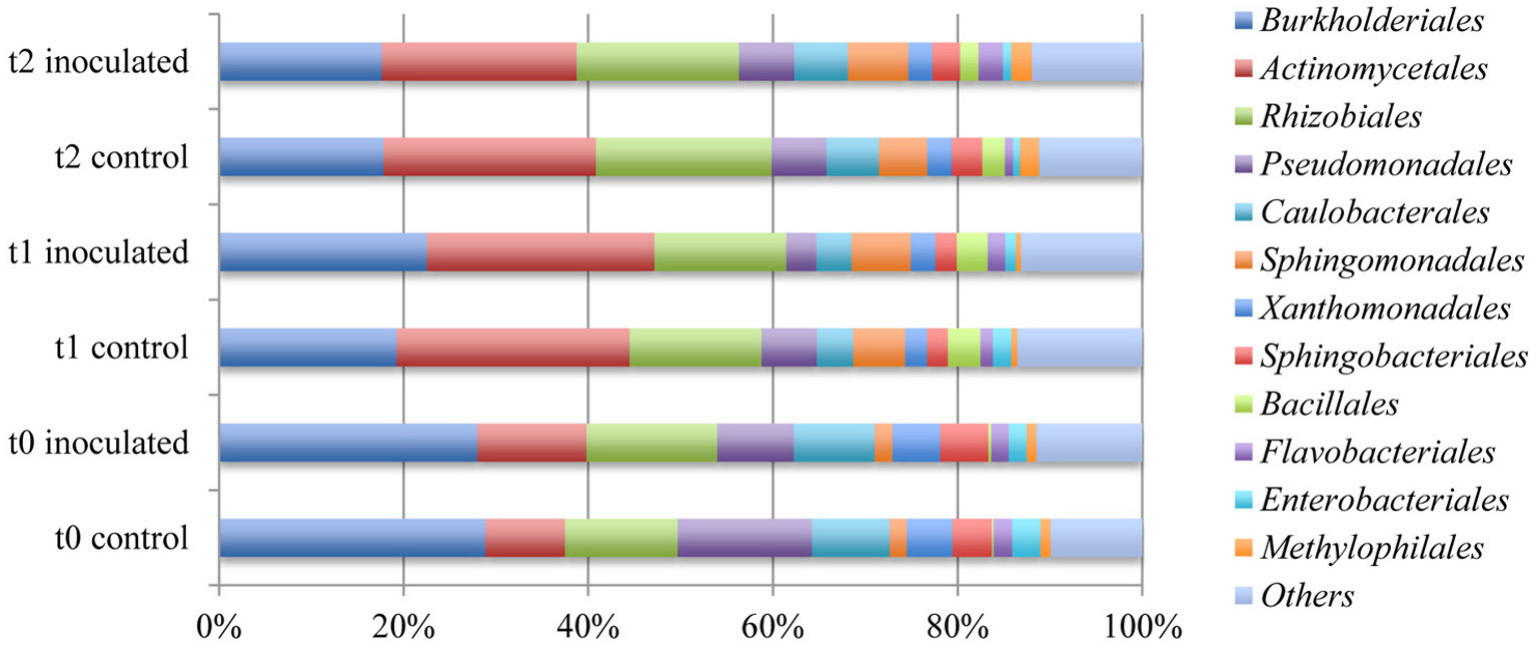
**Comparison of the ten most abundant orders within the rhizosphere of lettuce**. Abundances were calculated by means of comparison of metagenome sequence reads against the GenBank database within the MG-RAST software package.

Analysis of the most abundant orders revealed that *Burkholderiales, Actinomycetales, Rhizobiales*, and *Pseudomonadales* represented between 62% and almost 66% of the microbial communities in all six samples that where either inoculated or not treated (Table 5c and Figure 5). A decrease from around 28.5% annotated *Burkholderiales* in the t0 sample to an average of 19.3% in the other samples was noticeable. The ratios of annotated *Actinomycetales* increased from around 10% in the first samples to over 20% after 2 and 5 weeks of cultivation. The differences between samples collected at different time points were more distinct as compared to samples for different treatments.

The most obvious change probably caused by the application of strain FZB42 was the relative abundance of annotated *Pseudomonadales* varying between 14.5 and 8.3% in the samples taken at planting. The amount decreases to 6.0% in the control and 3.3% in the with FZB42 treated sample after 2 weeks and around 6% in both samples drawn after 5 weeks.

**Figure 6:**
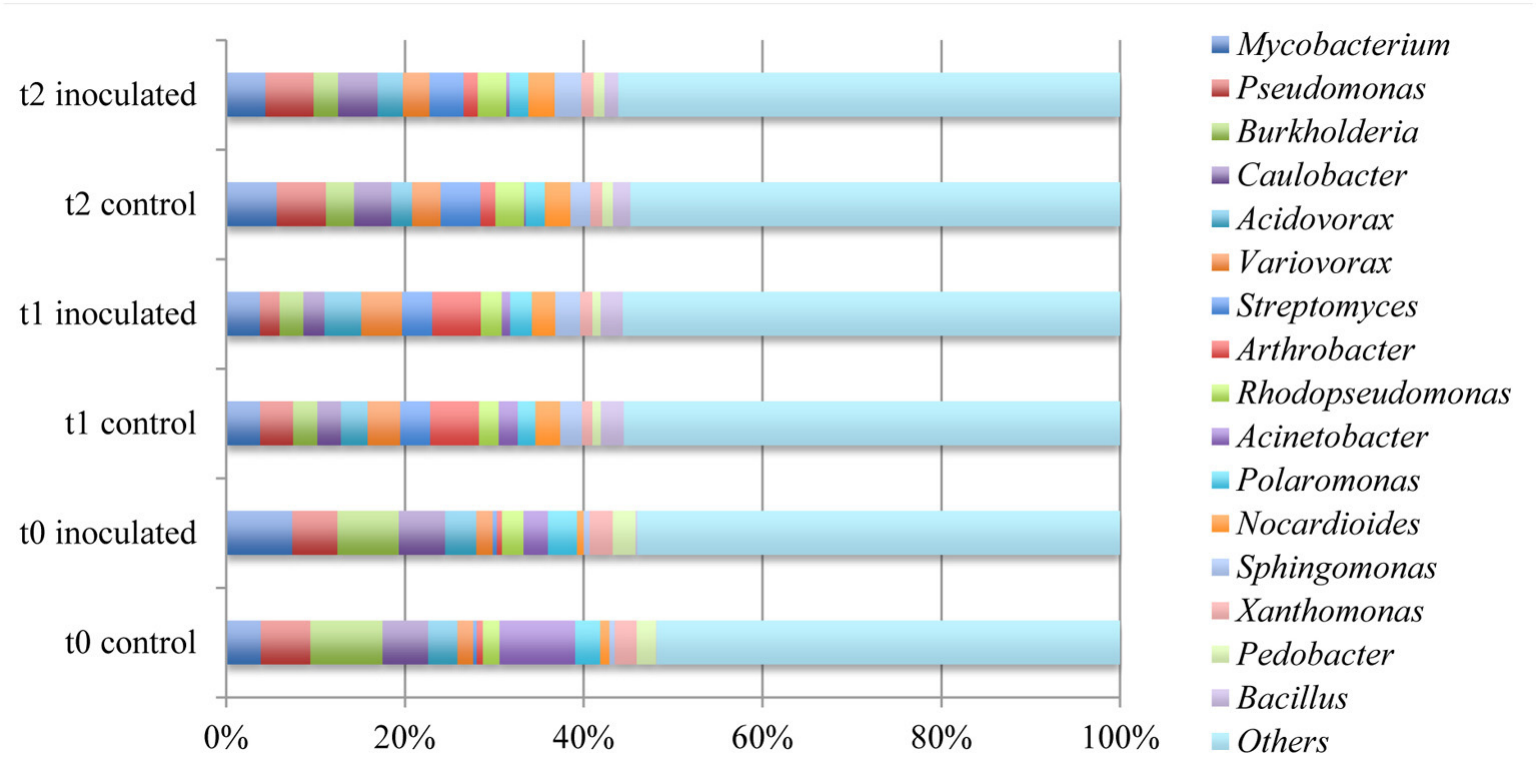
**Comparison of the ten most abundant genera within the rhizosphere of lettuce**. Abundances were calculated by means of comparison of metagenome sequence reads against the GenBank database within the MG-RAST software package.

**Figure 7:**
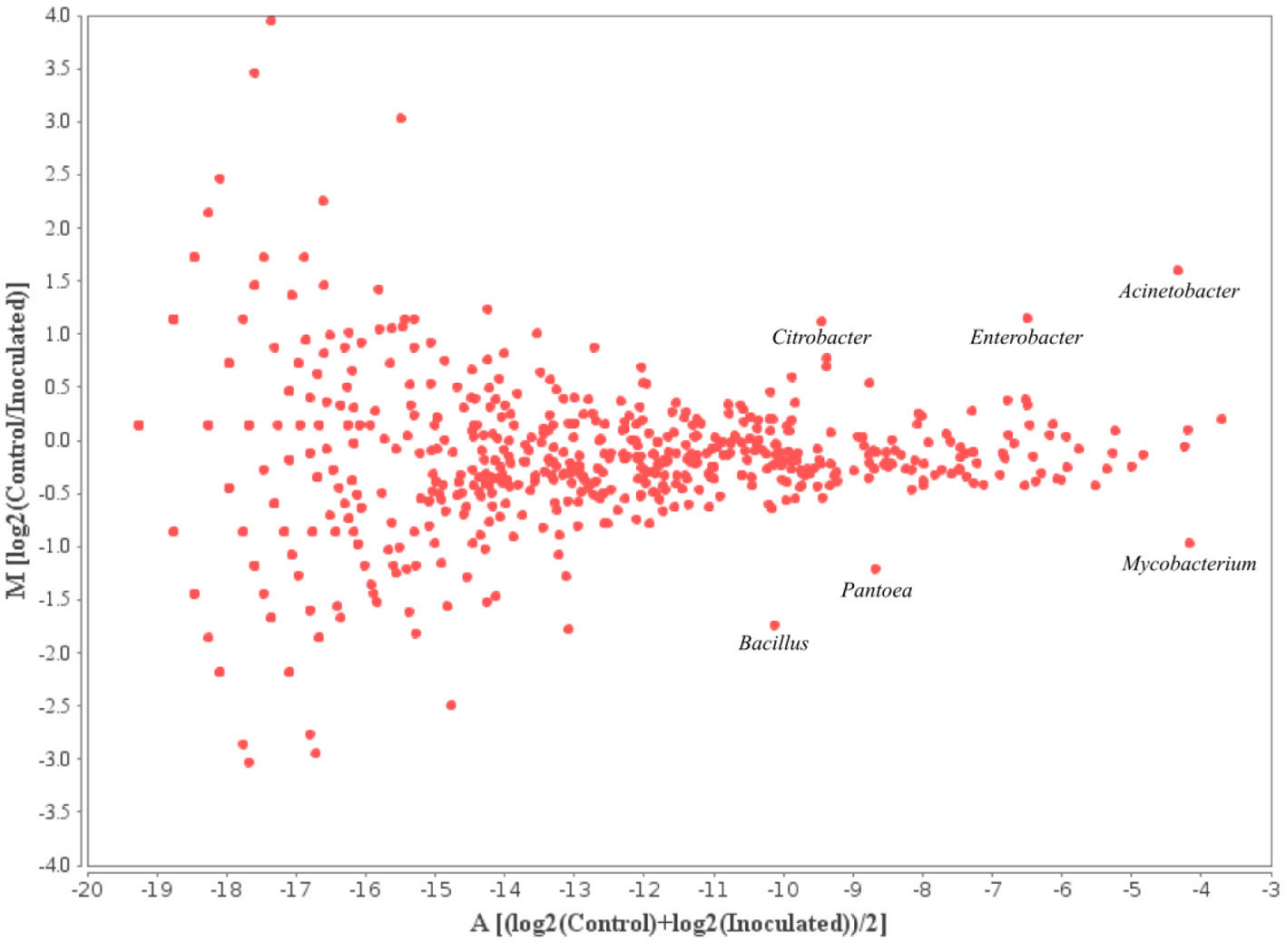
**MA-Plot visualizing the values of annotated genera within the t0 samples**. Log2(Control/Inoculated) indicates the fold-changes in the abundances of annotated genera between the two samples; (log2(Control)+log2(Inoculated))/2 shows the average abundance of an annotated genus.

**Figure 8:**
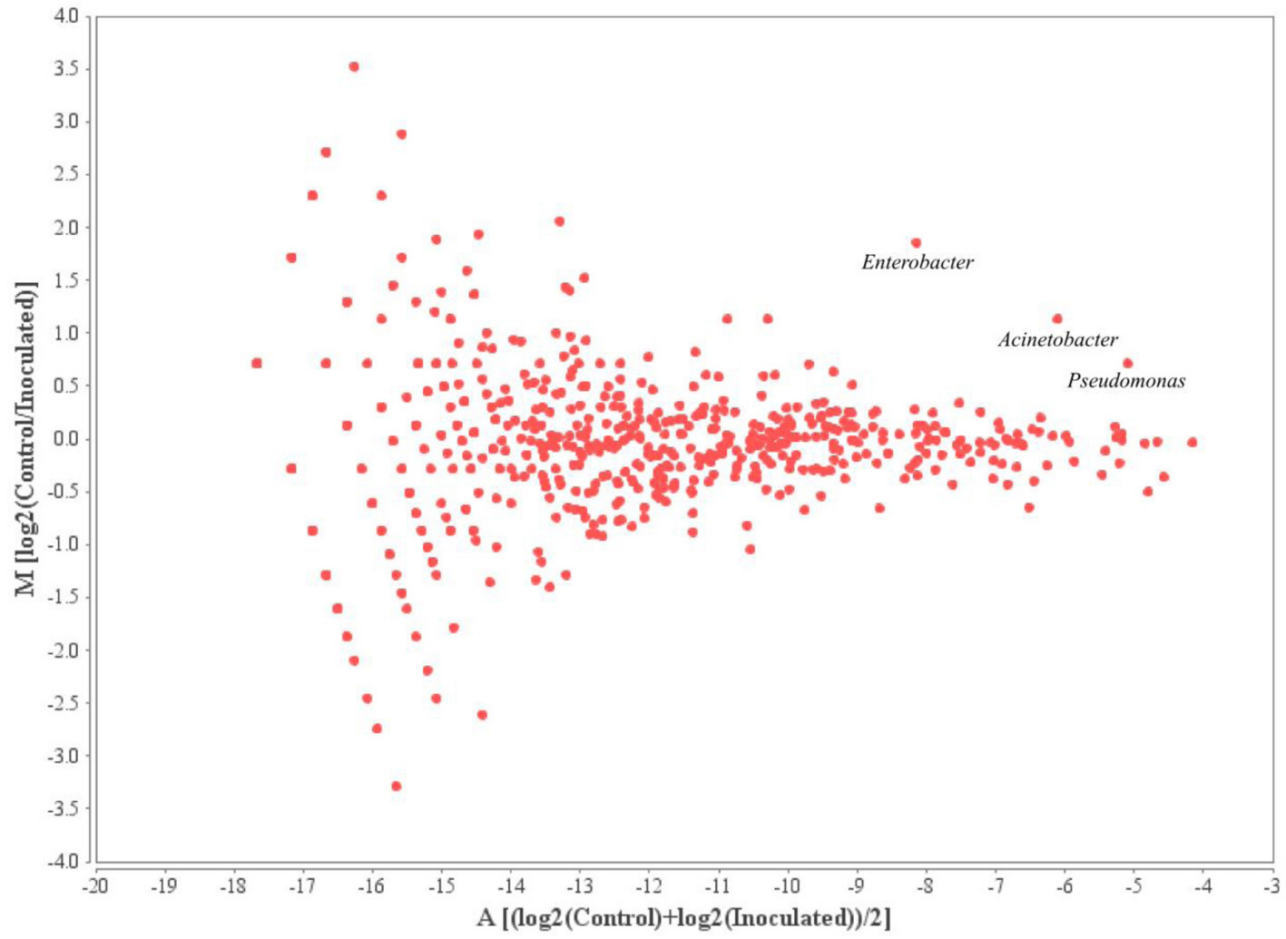
**MA-Plot visualizing the values of annotated genera within the t1 samples**. Log2(Control/Inoculated) indicates the fold-changes in the abundances of annotated genera between the two samples; (log2(Control)+log2(Inoculated))/2 shows the average abundance of an annotated genus.

**Figure 9:**
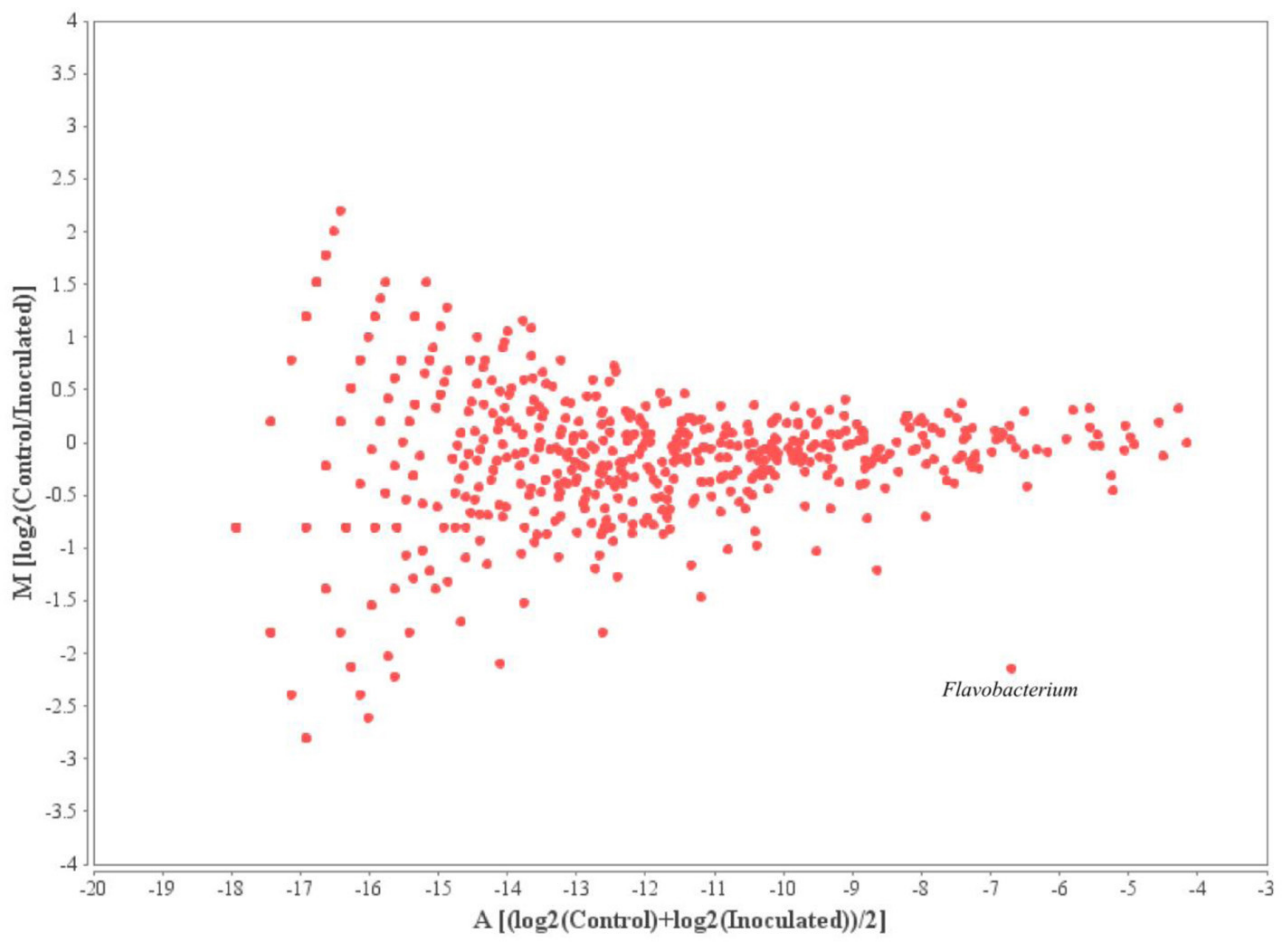
**MA-Plot visualizing the values of annotated genera within the t2 samples**. Log2(Control/Inoculated) indicates the fold-changes in the abundances of annotated genera between the two samples; (log2(Control)+log2(Inoculated))/2 shows the average abundance of an annotated genus.

The 10 most prominent genera within the samples taken from different time points add up to 16 genera representing around 45% of all annotated sequences. *Mycobacterium, Pseudomonas, Burkholderia*, and *Caulobacter* represented the most abundant genera within the microbial rhizosphere communities (Table 5d and Figure 6). Among the ratios for *Mycobacteria*, an increase from 3.9 to 7.4% was detectable for the control and the with FZB42 treated samples of t0, whereas the ratios were almost stable among the remaining samples that were either treated with FZB42 or not treated. Relative abundances for other genera revealed more pronounced changes between the samples that were taken at different time points than between the differentially treated samples. M/A plots of the annotated metagenome sequences on genus level visualized the distributions of detected genera clearly (Figures 7–9). The dense clouds around zero on the y-axis of these plots indicated a low fold-change in the abundances of annotated genera between the control and the inoculated samples. Higher variations were only noticeable within the genera that were annotated at very low abundances and were therefore of minor interest, because they do not contribute to the main share of the microbial communities. Within the t0 control sample a higher number of sequences were classified as *Acinetobacter*, *Enterobacter*, and *Citrobacter* in comparison to the inoculated sample. In the inoculated sample from this time point, the number of annotated *Bacillus, Pantoea*, and *Mycobacterium* was increased (Figure 7). The samples taken after 2 weeks of cultivation in the field showed even less noticeable differences (Figure 8). Only the abundances of *Acinetobacter* and *Enterobacter* were slightly increased in the control samples. Within the samples of t2 only the genus *Flavobacterium* was detected in a higher abundance in the inoculated sample (Figure 9).

Accordingly, application of *B. amyloliquefaciens* FZB42 appeared to have almost no effect on composition of the lettuce rhizosphere communities, whereas the growth states of the analyzed plants seem to alter the composition of the communities in a more noticeable manner.

### Analysis of the potential impact of *B. amyloliquefaciens* FZB42 on annotated functional subsystems of microbial community members in the lettuce rhizospheres

In order to gain further insights into community changes at the functional level, classifications of annotated genes according to functional subsystems as defined within MG-RAST of all samples were compared. The ratios of sequences assigned to any subsystem decreased from around 74.6% in the t0 samples to around 62% within the control samples and 57% within the inoculated samples taken after 2 and 5 weeks of cultivation in the field (Table 6). The main focus was put on the carbohydrate, nitrogen and amino acid metabolic pathways, since these subsystems are important for utilization and metabolism of plant root exudates.

**Table 6:** **Sequences assigned to all functional subclasses within the metagenome data sets calculated within the MG RAST analysis software with default settings**.

	**t0 control**	**t0 inoculated**	**t1 control**	**t1 inoculated**	**t2 control**	**t1 inoculated**
HQ sequences	1,869,056	2,446,824	783,195	1,470,887	1,550,845	971,074
Assigned sequences	1,419,743	1,791,952	485,868	837,363	967,685	557,903
	75.96%	73.24%	62.04%	56.93%	62.40%	57.45%

Examination of the relative amounts of metagenome sequences assigned to specific subsystems for different samples did not show any pronounced changes neither between with FZB42 treated or non-treated samples nor over the duration of the cultivation (see Table 7). Closer examination of the annotated genes within these mentioned functional subsystems of the samples also revealed very similar sets of assigned genes.

**Table 7:** **Relative distributions of functional subsystems calculated within the MG RAST analysis software with default settings**.

**Functional subsystems**	**t0 control**	**t0 inoculated**	**t1 control**	**t1 inoculated**	**t2 control**	**t2 inoculated**
Amino acids and derivatives	8.6	8.6	9.0	9.0	8.8	8.9
Carbohydrates	10.2	10.1	10.8	11.0	10.7	10.9
Cell division and cell cycle	1.3	1.3	1.4	1.3	1.4	1.3
Cell wall and capsule	3.8	3.7	3.5	3.4	3.5	3.5
Clustering-based subsystems	14.7	14.8	15.2	15.1	15.1	15.0
Cofactors, vitamins, prosthetic groups, pigments	6.6	6.7	6.8	6.8	6.9	6.9
DNA metabolism	4.2	4.3	4.1	4.1	4.1	4.1
Dormancy and sporulation	0.2	0.2	0.2	0.2	0.2	0.2
Fatty acids, lipids, and isoprenoids	3.1	3.2	3.2	3.1	3.2	3.2
Iron acquisition and metabolism	1.2	1.1	0.7	0.7	0.8	0.8
Membrane transport	3.5	3.4	3.2	3.1	3.2	3.2
Metabolism of aromatic compounds	2.1	2.0	2.0	2.0	2.0	2.1
Miscellaneous	8.4	8.4	8.4	8.3	8.3	8.3
Motility and chemotaxis	1.2	1.1	0.8	0.8	0.9	0.9
Nitrogen metabolism	1.2	1.2	1.2	1.2	1.3	1.3
Nucleosides and nucleotides	2.8	2.8	3.0	3.0	2.9	2.9
Phages, prophages, plasmids, transposable elements	1.5	1.6	1.5	1.5	1.4	1.4
Phosphorus metabolism	0.9	0.9	0.9	0.9	0.8	0.8
Photosynthesis	0.1	0.1	0.1	0.1	0.1	0.1
Potassium metabolism	0.5	0.5	0.4	0.4	0.5	0.4
Protein metabolism	7.6	7.7	7.5	7.7	7.6	7.5
RNA metabolism	4.1	4.1	3.9	3.9	3.9	3.9
Regulation and cell signaling	1.5	1.5	1.5	1.5	1.5	1.5
Respiration	3.4	3.4	3.6	3.5	3.5	3.6
Secondary metabolism	0.4	0.4	0.4	0.5	0.5	0.4
Stress response	2.7	2.7	2.6	2.7	2.7	2.7
Sulfur metabolism	1.3	1.3	1.3	1.3	1.3	1.3
Virulence, disease, and defense	3.0	3.0	2.8	2.8	2.8	2.8

Within the functional subclass of carbohydrate metabolism, some minor changes were detected between the samples taken at different time points (Table 8). Frequencies of detected genes associated with the metabolism of di-, oligo-, and polysaccharides increased slightly in the samples taken after 2 and 5 weeks of cultivation. Additionally, the frequencies of annotated genes involved in the metabolism of different organic acids decrease slightly from around 6.1% within the samples taken after 2 days to 5.5% for the samples taken after 2 and 5 weeks.

**Table 8:** **Relative abundances of annotated subsystems within the annotated functional system of carbohydrate metabolism**.

	**t0 control**	**t0 inoculated**	**t1 control**	**t1 inoculated**	**t2 control**	**t2 inoculated**
Central carbohydrate metabolism	29.7	30.1	30.1	30.4	30.0	30.3
Fermentation	12.8	13.4	13.1	12.6	12.8	12.7
One-carbon metabolism	11.6	11.8	11.9	12.1	11.8	11.7
Monosaccharides	11.2	10.5	10.0	9.8	10.3	10.3
Miscellaneous	8.8	8.5	8.5	8.5	8.6	8.5
Di- and oligosaccharides	8.1	7.8	8.5	8.7	8.5	8.8
Organic acids	6.2	6.0	5.5	5.6	5.5	5.5
CO_2_ Fixation	5.6	5.7	5.5	5.5	5.6	5.5
Sugar alcohols	2.9	2.8	3.3	3.3	3.2	3.1
Aminosugars	1.6	1.7	1.4	1.4	1.6	1.5
Polysaccharides	1.4	1.4	1.9	1.9	1.9	1.9
Predicted carbohydrate hydrolases	0.3	0.3	0.3	0.3	0.3	0.3

Frequencies of several annotated genes within the functional subclass of nitrogen metabolism are probably influenced by either the growth state of the plants or the transfer of plants into the field (Table 9). Genes involved in ammonia assimilation are more prominent within the first sample. Their frequencies decrease from around 47% at planting to values between 44 and 45% after 2 and 5 weeks. The ratios of genes within the annotated subsystem of nitrosative stress decreased in the course of the cultivation from 5.7% to around 5% and 4.4%. In contrast, frequencies of genes within the subsystem of nitrate and nitrite ammonification increased slightly during the course of the cultivation. Additionally, genes involved in nitrogen fixation increased in a similar manner.

**Table 9:** **Relative abundances of annotated subsystems within the annotated functional system of nitrogen metabolism**.

	**t0 control**	**t0 inoculated**	**t1 control**	**t1 inoculated**	**t2 control**	**t2 inoculated**
Ammonia assimilation	47.2	47.1	44.1	43.3	44.7	45.3
Nitrate and nitrite ammonification	21.0	19.8	22.1	21.4	22.3	22.4
Denitrification	8.5	8.4	7.4	8.2	6.9	7.1
Allatoin utilization	5.7	5.5	7.1	6.1	5.4	5.2
Nitrosative stress	5.7	5.7	5.1	5.0	4.6	4.2
Nitric oxide synthase	4.7	5.8	6.5	6.5	7.6	7.3
Dissimilatory nitrite reductase	3.2	3.3	3.0	3.7	3.5	3.5
Cyanite hydrolysis	3.2	3.4	3.1	3.5	3.6	3.2
Nitrogen fixation	0.4	0.7	1.2	1.7	1.2	1.4
Amidase cluster	0.3	0.3	0.2	0.3	0.3	0.4
Nitrilase	0.0	0.1	0.1	0.1	0.0	0.1

Within the functional subsystem of amino acid and amino acid derivative metabolism, the calculated frequencies of annotated subsystems almost showed no changes (Table 10). The most noticeable changes occurred in the subsystem of glutamine, glutamate, aspartate, asparagine and ammonia assimilation with frequencies increasing from around 10.3% to values around 11% in the samples taken after 2 and 5 weeks.

**Table 10:** **Relative abundances of annotated subsystems within the annotated functional system of amino acids and derivatives**.

	**t0 control**	**t0 inoculated**	**t1 control**	**t1 inoculated**	**t2 control**	**t2 inoculated**
Branched-chain amino acids	28.7	29.9	28.3	29.1	28.8	29.5
Lysine, threonine, methionine, and cysteine	21.4	21.7	21.2	21.4	21.3	21.4
Aromatic amino acids and derivatives	11.5	11.2	10.9	10.5	10.7	10.9
Arginine; urea cycle, polyamines	11.0	10.5	11.2	11.0	11.1	11.0
Glutamine, glutamate, aspartate, asparagine; ammonia assimilation	10.4	10.2	11.4	11.2	11.1	10.7
Alanine, serine, and glycine	9.8	9.6	10.2	10.0	10.1	10.0
Proline and 4-hydroxyproline	3.7	3.5	3.1	3.0	3.1	3.0
Histidine metabolism	2.9	2.7	2.8	2.7	2.7	2.7
Miscellaneous	0.7	0.7	1.1	1.1	1.1	1.0

Additionally, metagenome sequences assigned to the functional context “defense mechanisms” such as antibiotic resistances and transport systems for toxic compounds that may be indicative for microorganisms resistant to secondary metabolites produced by *B. amyloliquefaciens* FZB42 were analyzed (Table 11). The relative abundances of sequences corresponding to this subsystem varied between 2.8 and 3% of all assigned sequences within each sample. Examination of the subgroups of the subsystem “defense mechanisms” substantiates the conclusion that application of *B. amyloliquefaciens* FZB42 does not affect rhizosphere microorganisms featuring functions in the context of defense.

**Table 11:** **Relative abundances of annotated subsystems within the annotated functional system of Virulence, disease, and defense**.

	**t0 control**	**t0 inoculated**	**t1 control**	**t1 inoculated**	**t2 control**	**t2 inoculated**
Resistance to antibiotics and toxic compounds	80.7	81.4	78.9	78.8	79.7	79.8
–	9.7	9.1	10.3	10.2	10.4	10.2
Multi locus sequence typing	7.2	7.2	8.2	8.4	7.5	7.4
Adhesion	1.4	1.4	1.5	1.5	1.5	1.5
Bacteriocins, ribosomally synthesized antibacterial peptides	0.4	0.5	0.6	0.5	0.4	0.6
Invasion and intracellular resistance	0.4	0.3	0.3	0.3	0.3	0.3
Toxins and superantigens	0.1	0.1	0.2	0.2	0.2	0.1

In summary, comparison of the relative abundances of sequences assigned to sub-systems between samples neither showed pronounced differences between the inoculated and non-treated samples nor between the samples taken over the course of the field trial. Therefore, no major impact of the inoculant strain FZB42 on functional community profiles could be affirmed.

## Discussion

### *B. amyloliquefaciens* FZB42 is able to successfully colonize the lettuce rhizosphere in the course of the field experiment

Tracking of the inoculant strain *B. amyloliquefaciens* FZB42 in the lettuce rhizosphere in the course of the experiment was of crucial importance to estimate its competitiveness in this habitat and was achieved by mapping of metagenome sequences to the published reference genome of *B. amyloliquefaciens* FZB42. Corresponding fragment recruitments proved persistence of strain FZB42 in the rhizosphere. However, the number of mapped reads (contigs) and the number of covered *B. amyloliquefaciens* FZB42 genes decreased in the course of the plant cultivation indicating that the inoculant strain was less competitive compared to other community members. At the end of the experiment, strain FZB42 could still be traced at a clear level. Corrected and normalized values of the read numbers matching specific *B. amyloliquefaciens* FZB42 genes revealed that after 2 weeks 64% and after 5 weeks about 55% of the inoculant FZB42 was still traceable within the rhizosphere. These values are in accordance with previous results from reference experiments using a FZB42-derivative conferring rifampicin (Rif) resistance as inoculum. The CFUs per gram of soil of this strain decreased to 59% after 2 weeks and to 14% after 5 weeks of field cultivation (Chowdhury et al., 2013). The relatively high rate of recruited metagenome sequences from the non-inoculated samples is most probably due to the fact that sequences of rRNA genes and mobile genetic elements are very similar or even identical between different *Bacillus* species (Edwards et al., 2012). Accordingly, increasing numbers of mapped reads from the non-inoculated samples can be explained by the presence of other indigenous *Bacillus* species in the plant rhizosphere.

### Inoculation with *B. amyloliquefaciens* FZB42 has no major impact on the microbial communities in the lettuce rhizosphere and on the functional subsystem assignments of community members

Although competitiveness of *B. amyloliquefaciens* FZB42 in the lettuce rhizosphere is limited, application of the strain may affect composition of the indigenous microbial rhizosphere community, especially in early stages of plant development. Metagenomic sequencing and the subsequent comparison of deduced taxonomic profiles combining samples from several equally treated plant rhizospheres indicated that application of *B. amyloliquefaciens* FZB42 did not result in pronounced effects on the composition of the microbial communities of the lettuce rhizosphere. Microbial community changes seem more distinct between the samples drawn at different time points. Most obvious, changes in the composition of the microbial communities occur within the first 2 weeks of plant cultivation. It is known that plant root exudates have a major impact on the composition of microbial rhizosphere communities (Hartmann et al., 2009). Root exudation patterns change quantitatively and qualitatively during the growth of the plant and higher amounts of exudates were released into the rhizosphere usually at the initial growth phase as given after planting (Baudoin et al., 2002). Hence, changes in root exudate patterns may be responsible for a shift in microbial community structure in the course of plant growth. Similar studies showed that shifts within microbial rhizosphere communities are mainly due to different developmental stages of the plant (Inceoglu et al., 2013) and root exudates produced by the plants (Chaparro et al., 2013). In the study described here, community changes were most pronounced at the onset of cultivation whereas comparison of the taxonomic profiles between the samples after 2 and 5 weeks of plant growth revealed only minor differences in the ratios of identified taxa suggesting that environmental conditions in this time period are almost constant and therefore enable the establishment of an adapted bacterial community in the rhizosphere.

Comparison of the taxonomic profiles within the lettuce rhizosphere with other soil microbial communities shows similar compositions. Several studies on related habitats revealed the same five most abundant phyla *Acidobacteria*, *Bacteroidetes*, *Actinobacteria*, *Firmicutes*, and *Proteobacteria* with *Firmicutes* and *Proteobacteria* being the most prominent taxa in these microbial communities. Referenced studies include the analysis of the rhizosphere of *Lotus japonicus* (Unno and Shinano, 2013), the rhizosphere of sugarcane plants (Pisa et al., 2011) and the analysis of several soil metagenome samples *via* 16S rRNA gene amplicon sequencing (Vasileiadis et al., 2012).

Evaluation of assignments to functional subsystems of community members supported the results of the taxonomic classification. Similar to the taxonomic profiles, a shift over the course of the cultivation but not between the control and the with FZB42 treated samples was observed. Most obvious was the decrease within the ratio of sequences that could generally be assigned to any subsystem over the course of the study.

Changes within the functional subgroups that are associated with the metabolism of different saccharides, organic acids or amino acids are probably due to changes within the composition of root exudates from the lettuce plants (Baudoin et al., 2002). An additional factor for the slight decrease in the relative number of genes associated with the metabolism of organic acids after 2 and 5 weeks of cultivation probably was the transfer of plants into the field. The plants were grown in peat blocks that probably contained a higher amount of organic acids such as humic acids.

The detected changes corresponding to genes associated with nitrogen metabolism can probably be traced back to reactions of the lettuce plants to several abiotic stress factors (Molassiotis and Fotopoulos, 2011) such as reactive nitrogen species (RNS) including NO and other derivatives. The increase in the number of annotated nitrogen fixation genes may be ascribed to limited amounts of nitrogen in the soil and therefore the need to utilize atmospheric dinitrogen as nitrogen source. Additionally, nitrogen fixation has been proven to be a factor in the promotion of plant growth (Bulgarelli et al., 2013).

Although metagenome analysis is a very convenient method to determine the composition and functional profile of microbial rhizosphere communities only very few published studies applied this approach. Most of the earlier studies are based on T-RFLP and 16S rRNA gene amplicon sequencing which complicates direct comparison of the results obtained in this study. In addition to the inadequacy of comparing results obtained by applying different techniques, most studies only provide taxonomic profiles down to the level of orders. Therefore comparisons of microbial communities within different rhizospheres down to lower taxonomic levels are a relatively challenging task and demonstrate the need to perform further metagenome studies to get deeper insights in microbial communities associated with plants.

## Concluding remarks

Metagenome analyses of lettuce rhizosphere samples inoculated with *B. amyloliquefaciens* FZB42 vs. non-treated samples revealed that the inoculant strain only had a minor impact on the community structure within this habitat and on specific functional subsystems of community members. The fact that the number of sequences specifically matching the *B. amyloliquefaciens* FZB42 genome decreased during the growth period of lettuce indicated that the inoculant strain did not overgrow other relevant species within the lettuce rhizosphere.

In previously published studies, the described set of secondary metabolites produced by strain FZB42 was proven to mediate antibacterial and antifungal properties (Koumoutsi et al., 2004; Chen et al., 2006). These metabolites were suspected to induce changes within the microbial rhizosphere community, noticeable i.a., by shifts within the annotated functional subsystem of defense and resistance mechanisms. However, sequence analysis revealed only marginal changes within this subsystem so that it is likely that secondary metabolites do not have a major impact on phytopathogens in the rhizosphere and thus probably are not the key factor in the protection of the plant from pathogenic microorganisms. It should also be mentioned here that *B. amyloliquefaciens* FZB42 probably had not reached a critical density in the lettuce rhizosphere to exert its antibacterial and antifungal properties. Hence, other properties of the inoculant strain such as affecting the plant's ISR may have a more important impact. Studies addressing corresponding mechanisms should be performed to elucidate the mode of action of plant-growth-promoting rhizobacteria on the plant leading to prevention of infections by pathogens.

The results of this metagenome study suggest that the application of the commercially available inoculant strain FZB42 can be considered as a safe method to promote the health of the economically important lettuce plant and reduce severity of infections by phytopathogens like *R. solani*.

## Author contributions

The authors Magdalena Kröber, Daniel Wibberg, Rita Grosch, Soumitra P. Chowdhury, Felix Eikmeyer, and Bart Verwaaijen contributed to this work with either relevant experimental work, data analysis or writing, and revising the article critically for important intellectual content. The work was supervised by Andreas Schlüter, Anton Hartmann, and Alfred Pühler. All authors approved the final version to be published and agreed to be accountable for all aspects of the work in ensuring that questions related to the accuracy or integrity of any part of the work are appropriately investigated and resolved.

### Conflict of interest statement

This work was part of the PathControl cooperative project ‘Biological control of Bacillus amyloliquefaciens on the soil-borne fungal pathogen R. solani (FKZ 0315654B) funded by the BMBF and no conflict of interest needs to be declared. The authors declare that the research was conducted in the absence of any commercial or financial relationships that could be construed as a potential conflict of interest.
