# Supplementary material for: Effect of the strain Bacillus amyloliquefaciens FZB42 on the microbial community in the rhizosphere of lettuce under field conditions analyzed by whole metagenome sequencing
Source: Front Microbiol. 2014 May 27;5:252. doi: 10.3389/fmicb.2014.00252 (PMC4033844; doi:10.3389/fmicb.2014.00252)
Supplement: Supplementary file 1 [file DataSheet1.PDF]

Locus	gene name	Normalized number of mapped reads[a]			
		Control (Σ t0,t1,t2)	Inoculated t0	Inoculated t1	Inoculated t2
RBAM_000010	dnaA	0	2	0	0
RBAM_000040	recF	0	2	0	0
RBAM_000080	gyrA	1	0	1,66	0
RBAM_000120	guaB	0	2	0	0
RBAM_000170	glxK	1	0	4,97	0
RBAM_000180		0	0	4,97	0
RBAM_000220	yaaI	0	2	0	0
RBAM_000230	yaaJ	0	2	13,24	0
RBAM_000240		0	2	13,24	0
RBAM_000250	dnaX	0	1	0	0
RBAM_000290	bofA	0	2	0	0
RBAM_000300		0	2	0	0
RBAM_000310		8	18	1,66	2,51
RBAM_000360	yaaO	0	1	0	0
RBAM_000370	tmk	0	1	0	0
RBAM_000400	holB	0	2	0	0
RBAM_000470	metS	0	0	0	5,02
RBAM_000520	yabG	0	0	3,31	0
RBAM_000550	ispE	0	0	3,31	0
RBAM_000560	purR	0	0	3,31	0
RBAM_000640	mfd	0	2	0	0
RBAM_000660	yabM	0	2	0	0
RBAM_000700	yabQ	0	1	0	0
RBAM_000710	divIC	0	1	0	0
RBAM_000730	yabR	4	0	0	0
RBAM_000740		4	0	0	0
RBAM_000750	spoIIE	0	2	0	0
RBAM_000810	yacB	0	1	3,31	0
RBAM_000820	yacC	0	0	3,31	0
RBAM_000910	yacF	0	2	0	0
RBAM_000920		0	2	0	0
RBAM_000950	lysS	9	0	0	0
RBAM_000960		19	0	14,9	0
RBAM_000970		19	0	14,9	0
RBAM_000980		0	0	26,48	0

RBAM_000990		0	0	26,48	0
RBAM_001000		0	0	26,48	0
RBAM_001010		5	0	26,48	5,02
RBAM_001020		5	0	0	5,02
RBAM_001030		5	0	3,31	5,02
RBAM_001040		5	0	3,31	5,02
RBAM_001050		6	4	4,97	0
RBAM_001060		1	3	1,66	0
RBAM_001080	ctsR	0	2	0	0
RBAM_001090	mcsA	0	2	0	0
RBAM_001190	cysS	0	0	0	5,02
RBAM_001270	rplK	0	1	4,97	0
RBAM_001280	rplA	1	1	0	0
RBAM_001300	rplL	4	0	0	0
RBAM_001320	rpoB	23	0	0	5,02
RBAM_001330	rpoC	4	0	9,93	0
RBAM_001340	ybxF	0	1	0	0
RBAM_001350	rpsL	10	2	0	0
RBAM_001360	rpsG	4	0	0	2,51
RBAM_001370	fusA	6	0	8,28	12,56
RBAM_001380	tufA	38	35	9,93	22,61
RBAM_001400	rpsJ	1	0	0	0
RBAM_001410	rplC	2	0	0	7,54
RBAM_001420	rplD	0	2	0	0
RBAM_001430	rplW	0	2	0	0
RBAM_001440	rplB	1	0	0	0
RBAM_001450	rpsS	1	0	0	5,02
RBAM_001460	rplV	1	0	3,31	5,02
RBAM_001470	rpsC	2	0	3,31	2,51
RBAM_001480	rplP	6	0	11,59	2,51
RBAM_001490	rpmC	9	0	11,59	5,02
RBAM_001500	rpsQ	0	0	11,59	0
RBAM_001630	map	0	1	0	0
RBAM_001650	rpmJ	0	0	1,66	0
RBAM_001670	rpsK	0	0	1,66	0
RBAM_001700	ybxA	0	0	3,31	0
RBAM_001750	rpsI	2	3	0	0

RBAM_001760	ybaJ	0	3	0	5,02
RBAM_001800	ybaL	0	1	0	0
RBAM_001840	ybaN	4	0	0	0
RBAM_001850		0	2	0	0
RBAM_001900		0	0	0	10,05
RBAM_001940		0	0	0	10,05
RBAM_001950		0	0	0	10,05
RBAM_001960		0	0	0	10,05
RBAM_001970		0	0	21,52	0
RBAM_001980		1	0	1,66	0
RBAM_002010		3	0	0	0
RBAM_002120	feuA	0	2	0	0
RBAM_002130	ybbB	0	2	0	0
RBAM_002140	ybbC	0	2	0	0
RBAM_002220	ybbK	6	5	0	0
RBAM_002230		6	5	3,31	0
RBAM_002240		7	5	3,31	0
RBAM_002250		7	5	3,31	0
RBAM_002300	ybbT	0	0	0	5,02
RBAM_002460	ybxG	2	0	0	0
RBAM_002560	yqeB	0	2	0	0
RBAM_002570	ybyB	0	2	0	0
RBAM_002580	ybeC	0	2	3,31	0
RBAM_002590		0	2	0	0
RBAM_002630	yoaI	0	2	0	0
RBAM_002660	ybfG	0	2	0	0
RBAM_002670		0	2	0	0
RBAM_002790	ybgH	0	0	3,31	0
RBAM_002800	ybgJ	0	2	0	0
RBAM_003030	yccK	0	1	0	0
RBAM_003060	ycdC	0	2	0	0
RBAM_003070	ycdD	0	2	0	0
RBAM_003120	ycdI	0	2	0	0
RBAM_003130	yceA	0	2	0	0
RBAM_003220	opuAA	0	2	0	0
RBAM_003260	ycgA	0	2	0	0
RBAM_003290	ldh	0	2	0	0

RBAM_003300	mdr	0	2	0	5,02
RBAM_003540	nasA	0	0	3,31	0
RBAM_003650	srfAA	0	0	1,66	0
RBAM_003680	srfAC	0	1	0	0
RBAM_003690	srfAD	0	2	0	0
RBAM_003990	yclJ	0	1	0	0
RBAM_004000	yclK	0	1	0	0
RBAM_004040	yclM	0	0	3,31	0
RBAM_004090	ycnB	0	0	0	5,02
RBAM_004100	ycnC	0	2	0	0
RBAM_004150	gabT	0	0	3,31	0
RBAM_004200	ycnJ	0	0	1,66	0
RBAM_004210	ycnK	0	0	1,66	0
RBAM_004230	mtlA	0	1	0	0
RBAM_004320	kipA	0	1	0	0
RBAM_004340	ycsK	0	0	0	5,02
RBAM_004660	mntH	0	0	3,31	2,51
RBAM_004700	yvaD	0	0	3,31	0
RBAM_004760	ydbD	0	2	0	0
RBAM_004770	dctB	0	2	0	0
RBAM_004910	murF	0	4	0	0
RBAM_004970	ydcC	0	0	3,31	0
RBAM_005010	rsbR	0	0	3,31	0
RBAM_005110	ydcK	1	0	0	0
RBAM_005120		2	0	0	0
RBAM_005130		2	0	0	0
RBAM_005140		2	0	0	0
RBAM_005150		0	0	8,28	0
RBAM_005160		2	0	8,28	0
RBAM_005170		2	0	8,28	0
RBAM_005270	lrpA	4	2	0	0
RBAM_005280	yddJ	0	2	0	0
RBAM_005340	ywnB	1	0	1,66	0
RBAM_005350		1	0	3,31	2,51
RBAM_005400	cspC	3	0	0	5,02
RBAM_005450	yrkF	0	1	0	0
RBAM_005470	yrkI	0	2	0	0

RBAM_005480	yrkJ	0	2	0	0
RBAM_005550	yvdO	0	1	0	0
RBAM_005560		0	1	0	0
RBAM_005580	yvdP	0	3	0	0
RBAM_005890	adhB	0	2	0	5,02
RBAM_005900	yraF	0	2	0	5,02
RBAM_005910	yraG	0	0	0	5,02
RBAM_005930	ydfI	0	1	0	0
RBAM_005940	ydfJ	0	2	0	0
RBAM_005970	ynaD	0	0	3,31	0
RBAM_005980		0	0	3,31	0
RBAM_006020	ydgK	0	2	0	0
RBAM_006030	ydhB	0	2	0	0
RBAM_006070	yjhB	0	0	3,31	0
RBAM_006140	pbpE	0	0	3,31	0
RBAM_006150	ydhK	0	0	1,66	0
RBAM_006240	ydhU	0	2	0	0
RBAM_006280	ydjK	3	0	4,97	0
RBAM_006290		3	0	4,97	0
RBAM_006300		5	4	3,31	2,51
RBAM_006310		7	1	1,66	7,54
RBAM_006320		5	0	0	0
RBAM_006330		5	0	0	0
RBAM_006340		5	0	0	0
RBAM_006370	ydiC	0	1	0	0
RBAM_006380	ydiD	0	1	0	0
RBAM_006390	gcp	0	1	0	0
RBAM_006440	tatCy	0	2	0	0
RBAM_006480	groEL	2	0	1,66	0
RBAM_006580	pspA	0	1	0	0
RBAM_006590	ydjG	0	1	0	0
RBAM_006650	ydjL	0	0	3,31	0
RBAM_006700	cotA	0	0	1,66	0
RBAM_006750	yebA	0	2	0	5,02
RBAM_006760	guaA	0	1	0	0
RBAM_006770		0	2	0	0
RBAM_006780		0	2	0	0

RBAM_006860	purB	0	2	0	0
RBAM_006880	purS	0	2	0	0
RBAM_006890	purQ	0	2	0	0
RBAM_006900	purL	0	0	0	5,02
RBAM_006910	purF	0	0	6,62	0
RBAM_006920	purM	0	2	0	0
RBAM_006940	purH	0	1	0	0
RBAM_006950	purD	0	2	0	0
RBAM_006960	yerA	0	2	0	0
RBAM_007040	yerI	0	2	0	0
RBAM_007080	gatA	0	5	0	0
RBAM_007090	gatB	0	2	0	0
RBAM_007110	swrC	0	2	0	5,02
RBAM_007140	yefA	1	0	0	0
RBAM_007230	yeeI	2	0	0	0
RBAM_007250	cotJB	0	0	0	5,02
RBAM_007260	cotJC	0	0	0	5,02
RBAM_007350	yetJ	0	0	0	5,02
RBAM_007360	yetL	0	0	0	5,02
RBAM_007390	yetO	0	2	0	0
RBAM_007400		0	2	0	0
RBAM_007510	yfnI	0	0	0	2,51
RBAM_007530	yfnG	0	2	0	0
RBAM_007540	yfnF	0	2	0	0
RBAM_007570	yfnC	0	2	0	0
RBAM_007580	yfnA	0	2	0	0
RBAM_007630	yfmR	0	0	0	5,02
RBAM_007660	yfmM	1	0	3,31	0
RBAM_007720	pel	0	2	0	0
RBAM_007770	yflS	0	1	0	0
RBAM_007780	citS	0	0	1,66	0
RBAM_007870	yflI	0	2	0	0
RBAM_007880	yflH	0	2	0	0
RBAM_007980	treA	0	1	0	0
RBAM_008020	yfkO	0	1	0	0
RBAM_008030	yfkN	0	1	0	0
RBAM_008150	yfkE	0	0	3,31	0

RBAM_008160	yfkD	0	2	0	0
RBAM_008170	yfkC	0	2	0	0
RBAM_008200	pdaA	0	2	0	0
RBAM_008230	yfjQ	0	2	0	0
RBAM_008280	yfjN	0	2	0	0
RBAM_008360	glvA	0	0	3,31	0
RBAM_008400	yfiB	0	2	0	0
RBAM_008470	yfiQ	0	2	0	0
RBAM_008490		0	2	0	0
RBAM_008500	yfiT	0	2	0	0
RBAM_008600	yfhH	0	1	0	0
RBAM_008610	yfhI	0	1	0	0
RBAM_008660	yfhK	0	2	0	0
RBAM_008670	yfhL	0	2	0	0
RBAM_008690	csbB	0	3	0	0
RBAM_008780	ygaD	0	1	0	0
RBAM_008800	gsaB	0	1	0	0
RBAM_008850	ygxA	3	24	0	0
RBAM_008860		19	19	13,24	2,51
RBAM_008870		2	3	0	0
RBAM_008880		19	0	0	0
RBAM_008890		19	0	0	0
RBAM_008900		5	0	0	0
RBAM_008910		8	0	0	0
RBAM_008920		3	0	0	0
RBAM_008950		0	0	1,66	0
RBAM_008960		0	0	1,66	0
RBAM_009000		0	0	28,14	0
RBAM_009010		31	0	28,14	0
RBAM_009020		14	0	0	0
RBAM_009030		0	0	9,93	0
RBAM_009050	ygaJ	0	0	3,31	0
RBAM_009060	thiC	59	35	34,76	40,2
RBAM_009070	ygaK	0	6	0	0
RBAM_009170	ygaO	0	0	1,66	0
RBAM_009260	yhbH	1	0	0	0
RBAM_009290	yhcA	0	1	0	0

RBAM_009340	yhcG	0	2	0	0
RBAM_009350	yhcH	0	2	0	0
RBAM_009400	yhcL	0	0	3,31	0
RBAM_009510	yhcX	0	1	0	0
RBAM_009520	yhxA	0	3	0	0
RBAM_009550	glpK	0	1	3,31	0
RBAM_009560	glpD	0	0	0	2,51
RBAM_009590	yhcZ	0	0	1,66	0
RBAM_009600	yhdA	0	0	1,66	0
RBAM_009610	yhdB	0	0	1,66	0
RBAM_009620	yhdC	0	0	1,66	0
RBAM_009630	lytF	0	0	3,31	0
RBAM_009680	lytE	0	0	3,31	0
RBAM_009720	yhdG	0	2	0	0
RBAM_009730	yhdH	0	0	0	5,02
RBAM_009770	yhdO	0	2	0	0
RBAM_009820	yhdT	0	1	0	0
RBAM_009830	yhdU	0	1	0	0
RBAM_009890	yheN	0	2	0	0
RBAM_009930	nhaX	0	2	0	0
RBAM_009940	yheI	0	2	0	0
RBAM_010000	yheD	0	2	0	0
RBAM_010040	yhaZ	0	1	0	0
RBAM_010080	hemZ	0	0	3,31	0
RBAM_010140	yhaP	0	0	0	5,02
RBAM_010280	ecsA	0	2	0	0
RBAM_010290	ecsB	0	3	0	0
RBAM_010340	pbpF	0	5	0	0
RBAM_010350	hemE	0	0	3,31	0
RBAM_010370	hemY	0	2	0	0
RBAM_010540	yhfT	0	0	3,31	0
RBAM_010570	yhfW	0	2	0	0
RBAM_010630	comK	2	0	0	0
RBAM_010740	yhjN	0	2	0	0
RBAM_010790	addB	0	2	0	0
RBAM_010810	sbcD	0	1	0	2,51
RBAM_010820	yirY	0	2	0	0

RBAM_010830	yisB	0	2	0	0
RBAM_010910	yisK	0	2	0	0
RBAM_010960	yisP	0	2	0	0
RBAM_010970	yisQ	0	2	0	0
RBAM_011050	yitL	0	1	0	0
RBAM_011060		0	1	0	0
RBAM_011240	carB	0	1	0	0
RBAM_011310	comZ	0	2	0	0
RBAM_011320	yjzB	0	2	0	0
RBAM_011330	fabHA	0	2	0	0
RBAM_011340	fabF	0	2	0	0
RBAM_011350	yjaZ	0	1	0	0
RBAM_011360	appD	0	1	0	0
RBAM_011380	appA	0	2	0	0
RBAM_011630	yjbO	0	1	0	0
RBAM_011680	goxB	0	0	1,66	0
RBAM_011690	thiS	0	0	1,66	0
RBAM_011700	thiG	0	0	1,66	0
RBAM_011730	fabI	0	0	3,31	5,02
RBAM_011740	yjbX	0	0	3,31	0
RBAM_011880	blm	0	0	0	5,02
RBAM_011890		0	0	0	5,02
RBAM_011930	yjcI	1	0	0	0
RBAM_011940	yjcJ	1	0	0	0
RBAM_012020	yqcG	0	1	0	0
RBAM_012100	yfjF	1	0	0	0
RBAM_012160	lacE	0	2	0	0
RBAM_012180	lacG	0	2	0	0
RBAM_012270	yjgC	0	0	3,31	0
RBAM_012310	yjjA	0	0	1,66	0
RBAM_012400	exuR	0	2	0	0
RBAM_012480	yjqA	0	2	0	0
RBAM_012490	yjqB	0	2	0	0
RBAM_012570	xtrA	0	2	0	0
RBAM_012580	xpf	0	2	0	0
RBAM_012690	pit	0	0	3,31	0
RBAM_012700	ykaA	0	0	3,31	0

RBAM_012720	ykcA	0	1	0	0
RBAM_012730	ykcB	0	1	0	0
RBAM_012750	htrA	0	0	4,97	0
RBAM_012770	dppA	0	0	1,66	0
RBAM_012860	ykgB	0	2	0	0
RBAM_012870	ykgA	0	2	0	0
RBAM_013010	ohrB	0	1	0	0
RBAM_013030		0	2	0	0
RBAM_013060	ykoB	0	2	0	0
RBAM_013100	ydhD	0	0	3,31	0
RBAM_013110	ykzD	0	0	3,31	0
RBAM_013120	ykoK	0	2	0	0
RBAM_013130	tnrA	0	2	0	0
RBAM_013200	ykoX	0	1	0	0
RBAM_013260	ykrL	0	2	0	0
RBAM_013270	ykrM	0	2	0	5,02
RBAM_013280		0	2	0	0
RBAM_013300	ykrP	0	2	0	0
RBAM_013340	mtnK	0	1	0	0
RBAM_013350	ykrU	0	2	0	0
RBAM_013380	mtnX	0	1	0	0
RBAM_013390	mtnB	0	1	0	0
RBAM_013480	ykvI	0	0	1,66	0
RBAM_013560	ykvR	0	2	0	0
RBAM_013570		0	2	0	0
RBAM_013710	mcpC	0	4	0	0
RBAM_013760	ykuA	0	0	6,62	0
RBAM_013820	ykuC	0	0	0	5,02
RBAM_013830	ykuD	0	0	0	5,02
RBAM_014000	rok	0	0	1,66	0
RBAM_014010	yknT	0	0	1,66	0
RBAM_014020	mobA	0	0	0	5,02
RBAM_014080	yknW	0	2	0	5,02
RBAM_014090	yknX	0	2	0	0
RBAM_014110	yknZ	0	1	0	0
RBAM_014120	fruR	0	0	3,31	0
RBAM_014130	fruK	0	0	3,31	0

RBAM_014140	fruA	0	0	3,31	0
RBAM_014210	mreBH	0	2	0	0
RBAM_014330	mlnA	0	0	3,31	5,02
RBAM_014340	mlnB	0	1	3,31	5,02
RBAM_014350	mlnC	2	0	0	0
RBAM_014380	mlnF	0	3	0	0
RBAM_014390	mlnG	0	2	3,31	0
RBAM_014420	pdhA	0	0	1,66	0
RBAM_014470	slp	0	0	0	5,02
RBAM_014480		0	0	0	5,02
RBAM_014490	speA	0	0	3,31	5,02
RBAM_014520	ykzI	0	0	3,31	0
RBAM_014530	suhB	0	0	3,31	0
RBAM_014710	ftsW	0	2	0	0
RBAM_014720	pycA	0	2	0	0
RBAM_014750	ctaC	0	2	0	0
RBAM_014760	ctaD	0	2	0	0
RBAM_014770	ctaE	0	3	0	0
RBAM_014780	ctaF	0	1	0	0
RBAM_014820	ylbC	0	0	1,66	0
RBAM_014830	ylbD	0	0	1,66	0
RBAM_014930	ylbN	0	0	3,31	0
RBAM_014940	rpmF	0	0	3,31	0
RBAM_014950	ylbO	0	0	3,31	0
RBAM_014960	ylbP	0	0	3,31	0
RBAM_014970	ylbQ	0	2	0	0
RBAM_015020	pbpB	0	0	0	5,02
RBAM_015050	mraY	0	0	3,31	0
RBAM_015060	murD	0	2	3,31	0
RBAM_015070	spoVE	0	1	0	0
RBAM_015090	murB	0	3	0	0
RBAM_015100	divIB	0	1	0	0
RBAM_015130	bpr	0	0	3,31	0
RBAM_015140	spoIIGA	0	0	3,31	0
RBAM_015150	sigE	2	0	0	0
RBAM_015170	ylmA	0	2	0	0
RBAM_015200	ylmD	0	2	0	0

RBAM_015210	ylmE	0	1	0	0
RBAM_015220	ylmF	0	1	0	0
RBAM_015330	pyrC	1	2	0	0
RBAM_015340	pyrAA	1	0	0	7,54
RBAM_015370	pyrD	0	2	0	0
RBAM_015420	sat	0	2	0	0
RBAM_015480	yloB	0	0	3,31	0
RBAM_015510	gmk	0	2	0	0
RBAM_015530	yloI	0	1	0	0
RBAM_015540	priA	0	1	0	0
RBAM_015570	rsmB	0	2	0	0
RBAM_015580	yloN	0	2	0	5,02
RBAM_015590	prpC	0	0	0	5,02
RBAM_015610	engC	0	2	0	0
RBAM_015620	rpe	0	2	0	0
RBAM_015650	rpmB	0	2	0	0
RBAM_015660	yloU	0	2	0	0
RBAM_015670	yloV	0	2	0	0
RBAM_015770	smc	0	1	0	0
RBAM_015820	rpsP	2	0	3,31	0
RBAM_015870	rplS	3	0	0	0
RBAM_015920	sucC	0	0	3,31	0
RBAM_015990	clpY	0	1	0	0
RBAM_016000	codY	0	1	0	0
RBAM_016010	flgB	0	2	0	0
RBAM_016070	fliI	0	1	0	0
RBAM_016120	flgE	0	2	0	0
RBAM_016130		0	2	0	0
RBAM_016140	fliL	0	2	0	0
RBAM_016220	flhB	0	1	0	0
RBAM_016270	cheA	0	0	3,31	5,02
RBAM_016290	cheC	0	2	0	0
RBAM_016340	tsf	1	0	0	0
RBAM_016420	polC	0	1	3,31	0
RBAM_016440	nusA	0	2	0	0
RBAM_016510	ribC	0	0	3,31	0
RBAM_016570	spoVFA	0	2	0	0

RBAM_016590	asd	4	4	0	0
RBAM_016600	dapG	0	4	0	0
RBAM_016620	ymfA	0	2	0	0
RBAM_016630	tepA	0	2	0	0
RBAM_016640		0	1	0	0
RBAM_016650	ftsK	0	1	0	0
RBAM_016690	ymfC	0	2	3,31	0
RBAM_016700		0	1	0	0
RBAM_016710	ymfH	0	2	0	0
RBAM_016830	tdh	0	2	1,66	0
RBAM_016840	kbl	0	5	0	0
RBAM_016860	ymcA	0	2	0	0
RBAM_016980	baeJ	0	2	0	0
RBAM_016990	baeL	0	1	1,66	0
RBAM_017000	baeM	0	2	3,31	0
RBAM_017010	baeN	0	2	0	0
RBAM_017170	nrdI	0	0	6,62	0
RBAM_017180	nrdE	0	0	6,62	0
RBAM_017230	ynbA	0	0	0	10,05
RBAM_017240	ynbB	0	0	0	10,05
RBAM_017260	glnA	0	1	0	0
RBAM_017310		1	2	6,62	12,56
RBAM_017320	xynP	0	3	0	0
RBAM_017360	xylB	0	0	1,66	0
RBAM_017370	pps	8	4	1,66	7,54
RBAM_017490	yoaP	0	2	0	0
RBAM_017500	yoaO	0	0	3,31	0
RBAM_017590	yobO	0	0	0	5,02
RBAM_017710	yneF	2	0	3,31	0
RBAM_017730	ccdA	0	2	0	0
RBAM_017740	ccdB	0	2	0	0
RBAM_017800	citB	0	1	0	0
RBAM_017910	parC	0	2	0	5,02
RBAM_017940	alsT	0	2	3,31	0
RBAM_017950		0	0	0	2,51
RBAM_018020	yndG	0	2	3,31	0
RBAM_018030	yndH	0	2	3,31	0

RBAM_018040	yndJ	0	2	0	0
RBAM_018050		0	1	0	0
RBAM_018090		0	0	3,31	0
RBAM_018100	bglC	0	2	0	0
RBAM_018120	yvrG	1	0	0	0
RBAM_018170	bmyB	0	1	0	0
RBAM_018180	bmyA	0	4	3,31	0
RBAM_018220	scoA	0	2	0	0
RBAM_018230	yxjC	0	2	0	0
RBAM_018240	bioI	0	0	1,66	0
RBAM_018280	bioA	0	0	3,31	0
RBAM_018290	bioW	0	0	3,31	0
RBAM_018350	yngG	0	2	0	0
RBAM_018420	fenE	0	2	0	0
RBAM_018430	fenD	0	1	0	0
RBAM_018440	fenC	0	2	0	0
RBAM_018450	fenB	0	2	0	0
RBAM_018460	fenA	0	2	0	0
RBAM_018500	yoeB	0	2	3,31	0
RBAM_018510		0	2	0	0
RBAM_018520		0	2	0	0
RBAM_018540	ggt	2	0	0	0
RBAM_018550	ybcL	2	0	0	0
RBAM_018620	gltA	0	4	0	0
RBAM_018640	proJ	0	2	0	0
RBAM_018680	rtp	0	0	0	2,51
RBAM_018750	yoaC	0	0	1,66	0
RBAM_018760	yoaD	0	1	0	0
RBAM_018840	csaA	0	2	0	0
RBAM_018850	yobQ	0	2	0	0
RBAM_018940	yocD	0	1	0	0
RBAM_018950		0	1	0	0
RBAM_018980	yocL	0	2	0	0
RBAM_019090	yocR	0	2	0	0
RBAM_019200	yojI	0	2	0	0
RBAM_019220	yojG	0	2	0	0
RBAM_019280	yodA	0	2	0	0

RBAM_019290	yoaQ	0	2	0	0
RBAM_019360	bglA	0	2	1,66	2,51
RBAM_019370	yydK	0	2	0	0
RBAM_019380	ctpA	0	0	0	5,02
RBAM_019520	kamA	0	2	0	0
RBAM_019580	yosT	0	2	0	0
RBAM_019610	cgeC	0	2	0	0
RBAM_019640	phy	0	2	0	0
RBAM_019920	ilvA	0	1	0	0
RBAM_019970	thyB	0	3	0	0
RBAM_020040	bsaA	0	0	1,66	0
RBAM_020060	ugtP	0	2	0	0
RBAM_020070	cspD	0	2	0	0
RBAM_020180	ypbR	0	2	0	0
RBAM_020200	bcsA	0	2	0	0
RBAM_020360	yprB	0	0	3,31	0
RBAM_020370	yprA	0	2	0	0
RBAM_020660	ypjC	0	1	0	0
RBAM_020700	qcrC	0	1	0	0
RBAM_020710	qcrB	0	1	0	0
RBAM_020720	qcrA	0	2	0	0
RBAM_020820	trpC	0	2	0	0
RBAM_020830	trpD	0	2	0	0
RBAM_020880	cheR	0	2	0	0
RBAM_020900	hepT	0	2	0	0
RBAM_020920	hepS	0	2	0	0
RBAM_020940	mtrA	0	4	0	0
RBAM_020950	hupA	0	4	0	0
RBAM_020960	spoIVA	0	2	0	0
RBAM_021100	ypdA	0	2	0	0
RBAM_021110	gudB	0	2	0	0
RBAM_021120	mecB	0	2	0	0
RBAM_021330	dacB	0	2	0	0
RBAM_021340	ypuI	0	0	3,31	0
RBAM_021350	scpB	0	0	3,31	0
RBAM_021420	ribD	0	0	3,31	0
RBAM_021430	ypuD	0	2	0	0

RBAM_021460	ppiB	0	0	3,31	0
RBAM_021470	ypuA	0	0	3,31	0
RBAM_021490	spoVAF	0	1	0	0
RBAM_021580	spoIIAA	0	0	0	2,51
RBAM_021590	dacF	0	0	0	2,51
RBAM_021640	fur	0	2	0	0
RBAM_021680	mleA	0	2	0	0
RBAM_021690	mleN	0	2	0	0
RBAM_021800	yqkA	0	1	0	0
RBAM_021810	yqjZ	0	1	0	0
RBAM_021830	yqjW	0	2	0	0
RBAM_021860	yqzH	0	2	0	0
RBAM_021890	coaA	0	2	0	0
RBAM_021900	dsdA	0	0	1,66	0
RBAM_021950	dfnL	0	2	0	0
RBAM_021990	dfnH	0	0	3,31	0
RBAM_022000	dfnG	0	1	0	0
RBAM_022020	dfnE	0	2	0	0
RBAM_022030	dfnD	0	2	0	0
RBAM_022120	yqjM	0	2	3,31	0
RBAM_022170	yqjI	0	6	0	0
RBAM_022220	yqjD	0	2	0	0
RBAM_022310	bkdB	0	2	0	0
RBAM_022380	bkdR	0	0	3,31	0
RBAM_022410	mmgE	0	0	3,31	0
RBAM_022420	mmgD	0	0	3,31	0
RBAM_022460	yqiK	0	2	0	0
RBAM_022470	yqiI	0	2	0	0
RBAM_022550	spo0A	0	0	0	5,02
RBAM_022560	spoIVB	0	0	0	5,02
RBAM_022630	xseA	0	0	0	7,54
RBAM_022640	folD	0	0	0	7,54
RBAM_022780	efp	0	0	0	2,51
RBAM_022790	yqhT	0	0	0	2,51
RBAM_022800	yqhR	0	2	0	0
RBAM_022810	yqhQ	0	2	0	0
RBAM_022850	yqhM	0	2	0	0

RBAM_022860	yqhL	0	2	0	0
RBAM_022940	tasA	0	0	3,31	0
RBAM_023200	yqgO	0	0	3,31	0
RBAM_023210	yqgN	0	0	3,31	0
RBAM_023460	yqfQ	0	0	1,66	0
RBAM_023470	yqfP	0	0	1,66	0
RBAM_023480	yqfO	0	2	0	0
RBAM_023570	glyQ	0	2	0	0
RBAM_023580	recO	0	0	1,66	0
RBAM_023630	yqfF	0	0	1,66	0
RBAM_023640	phoH	0	2	0	5,02
RBAM_023650	yqfD	0	2	0	5,02
RBAM_023700	yqeY	0	0	3,31	0
RBAM_023710	rpsU	0	0	3,31	2,51
RBAM_023770	dnaK	0	2	0	5,02
RBAM_023810	lepA	0	2	13,24	0
RBAM_023830	spoIIP	0	1	0	5,02
RBAM_023840	gpr	0	1	0	0
RBAM_023850	rpsT	0	0	4,97	0
RBAM_023870	comEC	0	2	0	0
RBAM_023880	comEB	0	1	0	0
RBAM_023890	comEA	0	2	0	0
RBAM_023900	comER	0	2	0	0
RBAM_023960	aroD	0	0	3,31	0
RBAM_023970	yqeH	0	1	0	0
RBAM_023980	yqeG	0	1	0	0
RBAM_023990	sda	0	0	3,31	0
RBAM_024030	yqeD	0	1	0	0
RBAM_024040	spoIIIC	0	3	0	0
RBAM_024050		0	4	0	0
RBAM_024080	yrkQ	0	0	0	2,51
RBAM_024090	yrkP	0	1	1,66	2,51
RBAM_024160	yrdA	0	2	0	0
RBAM_024170		0	2	0	0
RBAM_024200	manP	0	0	1,66	5,02
RBAM_024210	manR	0	0	1,66	0
RBAM_024260	yrhJ	0	2	3,31	0

RBAM_024270	yrhI	0	0	3,31	0
RBAM_024350	yrhB	0	0	1,66	0
RBAM_024360	yrhA	0	0	1,66	0
RBAM_024370	mtn	0	0	0	5,02
RBAM_024380	yrrT	0	0	0	5,02
RBAM_024410	yrrR	0	1	0	0
RBAM_024440	yrrO	0	1	0	0
RBAM_024500	yrzL	0	3	0	0
RBAM_024510	alaS	0	3	0	0
RBAM_024590	yrrC	0	0	0	2,51
RBAM_024620	yrvO	0	2	0	5,02
RBAM_024630	yrzC	0	0	3,31	0
RBAM_024640	yrvN	0	5	3,31	0
RBAM_024720	apt	0	2	1,66	0
RBAM_024760	secDF	0	2	0	0
RBAM_024850	ruvA	0	0	0	5,02
RBAM_024910	nadC	0	2	0	0
RBAM_024920	nadB	0	2	0	0
RBAM_025030	spoIVFA	0	2	0	0
RBAM_025040	minD	0	0	1,66	0
RBAM_025050	minC	0	0	1,66	0
RBAM_025070	mreC	0	2	0	0
RBAM_025080	mreB	0	2	0	0
RBAM_025090	radC	0	1	0	0
RBAM_025130	folC	0	1	0	0
RBAM_025140	valS	0	1	0	2,51
RBAM_025150		0	2	0	0
RBAM_025160	ysxE	2	2	0	0
RBAM_025290	tig	0	2	0	0
RBAM_025300	ysoA	0	0	3,31	0
RBAM_025340	leuA	0	2	0	0
RBAM_025370	ilvB	0	0	0	15,07
RBAM_025390		0	6	0	0
RBAM_025400		0	6	0	0
RBAM_025440	rph	0	0	0	5,02
RBAM_025510	sdhA	0	0	4,97	0
RBAM_025580	etfA	0	0	3,31	0

RBAM_025650	mutSB	0	0	3,31	0
RBAM_025740	ysfB	0	1	0	0
RBAM_025750	ysfC	0	1	0	0
RBAM_025910	rplT	0	0	8,28	0
RBAM_025920	rpmI	0	2	0	0
RBAM_025930	infC	0	2	0	0
RBAM_025950	ysbA	0	2	0	0
RBAM_025960	lytT	0	2	0	0
RBAM_025970	lytS	0	2	0	0
RBAM_026010	ytxB	0	0	3,31	0
RBAM_026020	dnaI	0	0	3,31	0
RBAM_026050	speD	0	2	0	0
RBAM_026060	gapB	0	2	0	0
RBAM_026130	polA	0	1	0	0
RBAM_026140	phoR	0	1	0	0
RBAM_026160	mdh	0	3	0	0
RBAM_026170	icd	0	3	0	0
RBAM_026270	ytsJ	0	0	0	20,1
RBAM_026280	dnaE	0	0	0	5,02
RBAM_026290		0	1	0	0
RBAM_026300	ytrI	0	1	0	0
RBAM_026380	argG	0	1	0	0
RBAM_026410	ytxK	0	0	0	2,51
RBAM_026420	tpx	0	0	3,31	0
RBAM_026430	ytfJ	0	0	3,31	0
RBAM_026450	yteJ	0	0	0	5,02
RBAM_026460	sppA	0	0	0	5,02
RBAM_026520	nifZ	0	2	0	0
RBAM_026550	hisJ	0	0	0	5,02
RBAM_026560	yttP	0	0	0	5,02
RBAM_026590	rpsD	0	0	0	5,02
RBAM_026720	yraA	0	1	0	0
RBAM_026730		0	2	0	0
RBAM_026740		0	1	0	0
RBAM_026760		0	2	0	0
RBAM_026920	ytpT	0	2	3,31	0
RBAM_026960	ytoQ	0	1	0	0

RBAM_026990	malS	0	0	3,31	0
RBAM_027050	ytlQ	1	0	0	0
RBAM_027060	ytlP	1	0	0	0
RBAM_027070	ytkP	0	0	0	2,51
RBAM_027080	ytjP	0	0	0	2,51
RBAM_027090	ytiP	0	0	0	2,51
RBAM_027140	ytgP	0	0	3,31	0
RBAM_027150	ytfP	0	0	6,62	0
RBAM_027160	opuD	0	2	6,62	0
RBAM_027250	leuS	0	0	3,31	0
RBAM_027280	yttB	0	0	1,66	0
RBAM_027560	asnB	0	0	3,31	0
RBAM_027580	pckA	0	2	0	0
RBAM_027650	ytkC	0	2	0	0
RBAM_027670	ytkA	0	1	0	0
RBAM_027680	luxS	0	1	0	0
RBAM_027800	menD	0	2	0	2,51
RBAM_027840	ytaB	1	0	0	0
RBAM_027850		1	0	0	0
RBAM_027860		0	0	11,59	15,07
RBAM_027870		0	0	11,59	15,07
RBAM_027900		0	0	87,73	0
RBAM_027910		34	0	87,73	10,05
RBAM_027920		34	0	87,73	10,05
RBAM_027930		34	0	87,73	10,05
RBAM_027940		34	0	87,73	0
RBAM_027950		0	0	87,73	0
RBAM_027960		2	0	0	2,51
RBAM_027970		2	0	0	2,51
RBAM_027980		0	0	0	2,51
RBAM_028010		1	0	0	0
RBAM_028040		1	0	0	0
RBAM_028050		1	0	0	0
RBAM_028060		10	2	6,62	2,51
RBAM_028070		0	0	0	25,12
RBAM_028080		0	0	0	25,12
RBAM_028090		11	10	0	7,54

RBAM_028100	yuaJ	0	0	3,31	0
RBAM_028120	yuaF	0	2	0	0
RBAM_028130	yuaE	0	2	0	0
RBAM_028160	gbsA	0	2	0	5,02
RBAM_028210	hmp1	0	1	0	0
RBAM_028260	yubA	0	1	0	0
RBAM_028270	yulF	0	1	3,31	0
RBAM_028280		0	0	3,31	0
RBAM_028300	mcpA	0	2	0	0
RBAM_028310	tlpA	0	1	0	0
RBAM_028450	pgi	0	0	6,62	0
RBAM_028550	kinB	0	1	0	0
RBAM_028560	kapB	0	1	0	0
RBAM_028570	kapD	0	2	3,31	0
RBAM_028620	yufL	0	0	0	5,02
RBAM_028630	yufM	0	0	0	5,02
RBAM_028650	yufO	0	0	0	2,51
RBAM_028660	yufP	0	0	0	2,51
RBAM_028680	mrpA	0	0	3,31	0
RBAM_028710	mrpD	0	2	0	2,51
RBAM_028770	comP	0	1	0	0
RBAM_028820	yuxH	0	1	0	0
RBAM_028830	yueK	0	1	1,66	0
RBAM_028890	yueE	0	2	0	0
RBAM_028900	yueD	0	2	0	0
RBAM_028920	yueB	0	1	0	5,02
RBAM_028930	yukA	0	1	0	0
RBAM_029000	yukJ	0	0	0	2,51
RBAM_029010	dhbF	0	2	0	2,51
RBAM_029020	dhbB	0	1	0	0
RBAM_029030	dhbE	0	1	0	0
RBAM_029040	dhbC	0	2	0	0
RBAM_029050	dhbA	0	2	0	0
RBAM_029090	yuiF	0	2	0	0
RBAM_029100	yuiE	0	0	3,31	0
RBAM_029110	yuiD	0	2	3,31	0
RBAM_029120	yuiC	0	2	0	0

RBAM_029160	yumC	1	0	0	0
RBAM_029220	guaC	0	2	0	0
RBAM_029310	yutJ	0	0	3,31	0
RBAM_029320	yuzD	0	0	3,31	0
RBAM_029340	yuxL	0	0	3,31	0
RBAM_029390	yutG	0	2	0	0
RBAM_029450	yunB	0	0	0	5,02
RBAM_029500	pucH	0	2	0	0
RBAM_029510	pucR	0	1	0	0
RBAM_029590	pucF	0	2	0	0
RBAM_029610	yurJ	0	1	0	0
RBAM_029620	yurK	0	1	0	0
RBAM_029660	yurO	0	8	0	0
RBAM_029670	yurP	0	8	0	0
RBAM_029750	yurU	1	0	3,31	2,51
RBAM_029790	yurY	0	2	1,66	0
RBAM_029800	yurZ	0	0	1,66	0
RBAM_029910	yusK	0	2	0	0
RBAM_030030	yusT	2	0	0	0
RBAM_030040		2	1	0	0
RBAM_030110	yvtA	0	2	0	0
RBAM_030120	cssR	0	0	1,66	0
RBAM_030150	yuxN	0	2	0	0
RBAM_030160	citG	0	2	0	0
RBAM_030250	yvqH	0	1	0	5,02
RBAM_030260	yvqI	0	1	0	0
RBAM_030300	yvrB	0	0	0	5,02
RBAM_030310	yvrC	0	0	0	5,02
RBAM_030420	fhuG	0	1	0	0
RBAM_030430	fhuB	0	1	0	0
RBAM_030440	fhuD	0	0	0	5,02
RBAM_030450	yvsH	0	2	0	0
RBAM_030620	yvgS	0	0	3,31	0
RBAM_030670	yvgW	0	2	0	5,02
RBAM_030680	copA	0	2	0	0
RBAM_030790	ytmO	0	1	0	0
RBAM_030800	ytmN	0	1	0	0

RBAM_030890	smpB	0	0	1,66	0
RBAM_030910	yvaK	0	2	0	0
RBAM_030970	spaK	0	0	3,31	0
RBAM_030980	spaR	0	4	0	0
RBAM_030990	spaG	0	4	0	0
RBAM_031100	opuCA	0	2	0	0
RBAM_031300	gapA	2	0	3,31	0
RBAM_031320	araE	0	0	0	5,02
RBAM_031350	yvbU	0	1	0	0
RBAM_031510	sigL	0	3	0	0
RBAM_031520	yvfG	0	3	0	0
RBAM_031530	epsO	0	3	0	0
RBAM_031560	epsL	0	2	0	0
RBAM_031570	epsK	0	2	0	0
RBAM_031620	epsF	0	2	0	0
RBAM_031790	yveG	0	1	0	0
RBAM_031800		0	1	0	0
RBAM_031840	ywbF	0	2	0	0
RBAM_032160	yvoF	0	2	0	0
RBAM_032190	hprK	0	0	3,31	0
RBAM_032200	nagA	0	1	3,31	2,51
RBAM_032210	nagB	0	0	0	2,51
RBAM_032220	yvoA	0	2	0	0
RBAM_032240	yvnB	0	2	0	0
RBAM_032320	csbA	0	2	0	0
RBAM_032330	yvkC	0	2	0	0
RBAM_032340	yvkB	0	2	0	0
RBAM_032440	prfB	0	1	0	0
RBAM_032450	secA	0	0	0	5,02
RBAM_032500	fliD	0	1	0	0
RBAM_032510	hag	0	1	0	0
RBAM_032670	yvhJ	0	1	0	0
RBAM_032730	tuaD	0	4	0	0
RBAM_032740	tuaC	0	4	0	0
RBAM_032750	tuaB	0	2	0	0
RBAM_032780	lytB	0	0	0	2,51
RBAM_032810	mnaA	0	2	0	0

RBAM_032830	tagH	0	2	0	0
RBAM_032840	tagG	0	2	0	0
RBAM_032880	tagA	2	0	0	0
RBAM_032890	tagB	2	0	0	0
RBAM_032900	lytD	0	0	3,31	0
RBAM_032950	gerBC	0	2	0	0
RBAM_032960	ywtG	0	2	0	0
RBAM_033080	rbsR	0	2	0	0
RBAM_033170	alsS	0	2	0	0
RBAM_033220	cotH	0	0	3,31	0
RBAM_033280	ywrC	0	2	0	0
RBAM_033300	ywrA	0	2	0	0
RBAM_033460	ywqE	0	0	3,31	0
RBAM_033470	ywqD	0	0	3,31	0
RBAM_033480	ywqC	0	0	3,31	0
RBAM_033500	ywqA	0	2	0	0
RBAM_033610	mbl	0	0	0	5,02
RBAM_033620	spoIIID	1	0	3,31	5,02
RBAM_033650	ywoE	0	0	0	5,02
RBAM_033660	ywoD	0	0	1,66	0
RBAM_033710	ywoA	0	2	0	0
RBAM_033720	ywnJ	0	2	0	0
RBAM_033810	ureC	0	1	3,31	0
RBAM_033850	ywmF	0	0	0	5,02
RBAM_033860	rapB	0	0	0	5,02
RBAM_033920	murAA	0	4	0	0
RBAM_033930	ywmB	0	4	0	0
RBAM_033990	atpA	0	0	0	5,02
RBAM_034000	atpH	0	2	0	0
RBAM_034050	upp	0	0	0	5,02
RBAM_034170	prfA	0	0	0	2,51
RBAM_034210	ywkA	0	2	0	0
RBAM_034270	tal	0	1	3,31	0
RBAM_034280	fbaA	1	1	3,31	0
RBAM_034350	uvsE	0	0	3,31	0
RBAM_034430	narG	3	0	0	0
RBAM_034550	phrF	0	2	0	0

RBAM_034560		0	2	0	0
RBAM_034640	ywhE	0	1	0	0
RBAM_034650	ywhD	0	1	0	0
RBAM_034670	ywhB	0	0	3,31	0
RBAM_034680	ywhA	0	2	3,31	0
RBAM_034770	rsfA	0	2	0	0
RBAM_034800		0	0	0	5,02
RBAM_034810		0	0	0	5,02
RBAM_034890	bacE	0	2	0	0
RBAM_034900	bacD	0	2	0	0
RBAM_034970	rocB	0	2	0	0
RBAM_034990	rocG	0	2	0	0
RBAM_035070	spsK	0	2	0	0
RBAM_035130	spsD	0	1	3,31	0
RBAM_035140	spsC	0	1	0	0
RBAM_035180	ywdK	0	2	0	0
RBAM_035190	ywdJ	0	2	0	0
RBAM_035320	vpr	0	2	0	0
RBAM_035360	nfrA	0	0	3,31	0
RBAM_035370	rodA	0	0	3,31	0
RBAM_035440	ydaS	0	2	0	0
RBAM_035450	galT	0	2	0	0
RBAM_035520	ywbO	0	2	0	0
RBAM_035530	ywbN	2	0	0	0
RBAM_035580	ywbH	1	0	0	0
RBAM_035620	ywbE	0	2	0	0
RBAM_035650	ywbA	0	2	0	0
RBAM_035660	epr	0	1	0	0
RBAM_035670	gspA	0	1	0	0
RBAM_035690	menA	0	0	3,31	0
RBAM_035750	ywaA	0	0	3,31	0
RBAM_035760	licH	0	0	4,97	0
RBAM_035820	yxlJ	0	0	3,31	0
RBAM_035840		0	0	0	2,51
RBAM_035890	ydhO	0	2	0	0
RBAM_035900	ydhP	0	2	0	0
RBAM_035930	ydhT	0	0	1,66	0

RBAM_035960	yxlA	0	0	3,31	0
RBAM_035970	yxkO	0	0	3,31	0
RBAM_036000	cydB	0	1	0	0
RBAM_036020	cimH	0	0	0	5,02
RBAM_036050	yxkF	0	2	0	0
RBAM_036110	yxjI	0	0	0	5,02
RBAM_036170	katE	0	2	0	0
RBAM_036220	dbpA	0	0	1,66	0
RBAM_036270	yxiK	0	1	0	0
RBAM_036280	yxiG	0	1	0	0
RBAM_036340	yxiE	0	0	1,66	0
RBAM_036350	bglH	0	0	1,66	0
RBAM_036370	bglP	0	0	3,31	0
RBAM_036440	hutG	0	2	0	0
RBAM_036460	pdp	0	2	0	0
RBAM_036470	nupC	0	2	0	0
RBAM_036630	yxdJ	0	0	0	2,51
RBAM_036640	mrsK2	0	2	0	0
RBAM_036730	iolF	0	0	0	2,51
RBAM_036740	iolE	0	2	0	0
RBAM_036760	iolC	0	2	0	0
RBAM_036900	yxaL	0	2	0	0
RBAM_036930	yxaJ	0	2	0	0
RBAM_036940	yxaI	0	1	0	0
RBAM_036950		0	0	3,31	0
RBAM_036970	ahpF	0	0	3,31	0
RBAM_036980		0	1	0	0
RBAM_037200	yycO	0	1	0	0
RBAM_037260	rocR	0	0	3,31	0
RBAM_037270		0	1	0	0
RBAM_037360	yycJ	0	1	0	0
RBAM_037370	yycI	0	1	0	0
RBAM_037410	yycF	0	1	0	0
RBAM_037420		0	14	0	0
RBAM_037430		0	14	0	0
RBAM_037460	yycE	0	14	0	0
RBAM_037470	dnaC	0	2	0	0

RBAM_037530	yycA	0	2	0	0
RBAM_037630	ppaC	2	4	0	0
RBAM_037670	yveB	0	0	1,66	0
RBAM_037690	yfmI1	0	2	0	0
RBAM_037700	yfm2	0	2	0	0
RBAM_037900	yyaL	0	2	0	0
RBAM_037930	yyaH	0	2	0	0
RBAM_037950	exoA	0	3	0	0
RBAM_037960	adaB	0	3	0	0
RBAM_038110	gidA	0	0	0	2,51
RBAM_038120	trmE	0	0	4,97	0
RBAM_038160	rpmH	0	0	3,31	0

a normalized to the largest metagenome sequence data set
